# Tissue material properties and computational modelling of the human tibiofemoral joint: a critical review

**DOI:** 10.7717/peerj.4298

**Published:** 2018-01-25

**Authors:** Abby E. Peters, Riaz Akhtar, Eithne J. Comerford, Karl T. Bates

**Affiliations:** 1 Department of Musculoskeletal Biology, Institute of Ageing and Chronic Disease, University of Liverpool, Liverpool, UK; 2 Department of Mechanical, Materials and Aerospace Engineering, School of Engineering, University of Liverpool, Liverpool, UK; 3 Institute of Veterinary Science, University of Liverpool, Liverpool, UK

**Keywords:** Ligaments, Bone, Finite element, Cartilage, Human knee, Material properties

## Abstract

Understanding how structural and functional alterations of individual tissues impact on whole-joint function is challenging, particularly in humans where direct invasive experimentation is difficult. Finite element (FE) computational models produce quantitative predictions of the mechanical and physiological behaviour of multiple tissues simultaneously, thereby providing a means to study changes that occur through healthy ageing and disease such as osteoarthritis (OA). As a result, significant research investment has been placed in developing such models of the human knee. Previous work has highlighted that model predictions are highly sensitive to the various inputs used to build them, particularly the mathematical definition of material properties of biological tissues. The goal of this systematic review is two-fold. First, we provide a comprehensive summation and evaluation of existing linear elastic material property data for human tibiofemoral joint tissues, tabulating numerical values as a reference resource for future studies. Second, we review efforts to model tibiofemoral joint mechanical behaviour through FE modelling with particular focus on how studies have sourced tissue material properties. The last decade has seen a renaissance in material testing fuelled by development of a variety of new engineering techniques that allow the mechanical behaviour of both soft and hard tissues to be characterised at a spectrum of scales from nano- to bulk tissue level. As a result, there now exists an extremely broad range of published values for human tibiofemoral joint tissues. However, our systematic review highlights gaps and ambiguities that mean quantitative understanding of how tissue material properties alter with age and OA is limited. It is therefore currently challenging to construct FE models of the knee that are truly representative of a specific age or disease-state. Consequently, recent tibiofemoral joint FE models have been highly generic in terms of material properties even relying on non-human data from multiple species. We highlight this by critically evaluating current ability to quantitatively compare and model (1) young and old and (2) healthy and OA human tibiofemoral joints. We suggest that future research into both healthy and diseased knee function will benefit greatly from a subject- or cohort-specific approach in which FE models are constructed using material properties, medical imagery and loading data from cohorts with consistent demographics and/or disease states.

## Introduction

The knee joint is a primary component of the musculoskeletal system that aids the absorption and transition of weight bearing forces. As an integral part of biomechanical movement the knee joint is often subjected to injury or disease such as ligament rupture (Mullaji et al., 2008; Hill et al., 2005), meniscal tears (Lange et al., 2007) and osteoarthritis (OA) (Zhang & Jordan, 2008). OA is one of the most common musculoskeletal conditions in the elderly population causing structural degeneration of tissues and ultimately leading to a decline in function (Rousseau & Garnero, 2012). The most common type of OA exists in the knee joint which is the leading cause of locomotor disability (Zhang & Jordan, 2008). The disease is encouraged by heredity influence, ageing, gender, obesity and trauma or injury to the affected joint (Manninen et al., 1996), known as secondary OA, and can often lead to joint replacement (Nigg & Herzog, 2006). Where the cause of the disease is unknown this is referred to as primary OA (Buckwalter & Martin, 2006). It is approximated that 40% of adults over the age of 70 will be affected by OA of the knee in the United States of America (Punzi, Oliviero & Ramonda, 2010), with direct lifetime medical costs of $12,400 per person (Losina et al., 2015). OA does not just present with direct joint degeneration but is intrinsically linked to other diseases and neuromuscular complications which can further exacerbate age-related issues such as sarcopenia and a loss of movement control. Individuals with OA have increased variability of gait spatial–temporal parameters (Kiss, 2011) which in turn can decrease locomotor stability and increase the risk of falls (Lord, Lloyd & Li, 1996; Hausdorff, Rios & Edelberg, 2001; Owings & Grabiner, 2004; Brach et al., 2005; Hollman et al., 2007).

Typically, research surrounding OA focuses on the deterioration of articular cartilage; however recent studies have highlighted the need to consider structural changes of subchondral bone in the progression of OA (Nigg & Herzog, 2006). Significant relationships have been identified between changes occurring in different tissues specifically observing molecular crosstalk (Lories & Luyten, 2011; Mahjoub, Berenbaum & Houard, 2012). OA is therefore more recently seen as a disease of the entire joint with biochemical and biomechanical factors influencing the progression and status of the disease. Each tissue has a specific role and functionality within the knee joint in order to aid movement and stability. Individual tissues have a distinct structure and material properties that define its adaptive and responsive behaviour in accordance with the biomechanics of movement (Punzi, Oliviero & Ramonda, 2010). Biochemical and mechanical changes naturally occur during ageing even in the absence of clinically defined injury or disease and these changes have been shown to modify form–function relationships at the knee joint (Hansen, Masouros & Amis, 2006); however, data is limited.

In order to fully understand the onset and progression of OA it is essential to characterise the basic relationships between structure and function within a healthy human knee and how tissues age in the absence of disease. Understanding biomechanics of anatomically complex structures like the knee joint is challenging particularly in humans where experimental approaches must largely be non-invasive. The difficulty of achieving direct quantitative measures of tissue behaviour together with more widespread availability of imaging technology (i.e. magnetic resonance imaging (MRI), X-ray computed tomography (CT)) has led to an increasing use of computational approaches, notably finite element (FE) analysis, to study knee joint form and function (Peña et al., 2005, 2006; Wang, Fan & Zhang, 2014). Once suitably validated such FE models may potentially circumvent the issues surrounding direct invasive measurement of tissue mechanics by producing quantitative predictions of the mechanical and physiological behaviour of multiple tissues simultaneously, thereby inherently calculating tissue interaction. This could be particularly useful in identifying tissue interaction that may occur during ageing and in the presence of disease.

Through use of parameterisation, models can also be used in a predictive capacity to address questions that cannot ethically or even practically be asked by experimentation on humans or animals. Specifically, iterations of the same model can be generated where aspects of structure including gross anatomy and material properties, and loading behaviour are non-invasively manipulated to quantify the impact on function. In this way parameterisation enables cause–effect relationships between anatomy and mechanics to be identified, whilst allowing the impact of individual and combinations of morphological characteristics to be isolated (Li, Lopez & Rubash, 2001). Model manipulations can also be used for testing surgical interventions, treatment strategies and prosthetics (Baldwin et al., 2012; Tuncer et al., 2013).

Models are by definition abstractions of reality and their constituent parts or input parameters are typically tailored to address a specific research question or hypothesis. Consequently models of the same anatomical structure, such as the knee joint, may vary considerably between studies according to the research objective. In the context of the human knee, for example it is common for researchers to use models to answer questions on one specific tissue (e.g. ligament injuries under specific stress and strain) and as such effort and complexity is invested in these specific tissues while it is deemed sufficient to invest less towards input values for other tissues (i.e. therefore simplifying cartilage representation to a linear elastic material, or bone treated as a rigid body). However, tissues within a joint inherently interact and behaviour of one is influenced by others, although to what extent to which tissues interact has not extensively been studied.

Subject specific FE modelling is useful in the application of OA as it can investigate the true interaction between multiple tissues and how changes in one can lead to implications in an adjacent tissue, which may lead to disease initiation or progression. For example, ligament ruptures are histologically known to occur in the presence of OA (Mullaji et al., 2008), yet the impact or causative link to cartilage degeneration is unknown. Whilst efforts have been made to investigate this disease through computational approaches, it is indeed clear that there is a lack of baseline healthy measurements providing a foundation for comparative analyses. Research into the material properties of young healthy tissues surrounding the human knee is needed to compare to other cohort-specific groups. In the context of joint biomechanics this is crucial to understanding how, for example component parts of the joint function so that corrective therapeutics can restore joint function to the normal baseline as per the healthy sample measurements. Baseline healthy measurements are also crucial for basic science contexts such as sports biomechanics, where increasing biomechanical function is directly linked to performance. The accuracy of computational modelling approaches in general has been shown repeatedly to rely on good input data (Guo, Maher & Spilker, 2013; Kazemi, Dabiri & Li, 2013; Freutel et al., 2014). Direction of future research towards understanding the influence of donor age and ‘healthy’ versus pathological conditions on material properties with these new techniques has been cited as a key goal (Lewis & Nyman, 2008), but it is presently unclear of extent to which this has been achieved in the context of the human knee joint.

Evidently the human knee joint is crucial in biomechanical movement and function and has therefore the relevant literature has been reviewed extensively in recent years. Specifically, several reviews have discussed computational modelling of individual tissues of the knee joint. For example, Wilson et al. (2005) reviewed articular cartilage representations of behavioural and injury mechanisms, whilst Taylor & Miller (2006) reviewed both micro- and macro-level representation of cartilage tissue. Computational modelling of ligaments has also been reviewed by Woo, Johnson & Smith (1993) and Weiss & Gardiner (2001) focusing on viscoelasticity and one-dimensional to three-dimensional (3D) representations respectively. Whole knee joint modelling has also been reviewed in recent years by Peña et al. (2007a), Elias & Cosgarea (2007) and Kazemi, Dabiri & Li (2013). Whilst these reviews focused on advances in modelling, to date no review paper has critically evaluated the nature of material property available for human knee joint tissues and subsequently how this data has been transferred to FE models, with particular reference to ageing and OA.

The aim of this review paper is two-fold. Firstly, to conduct a review of scientific literature to understand what material property data currently exists for cartilage, bone and ligament samples from the human knee joint in an attempt to understand alterations during healthy ageing and disease status. Secondly, this paper aims to determine how this data has been subsequently applied within biomedical engineering in the form of existing FE models of the whole human knee joint. In doing so we collate a comprehensive database of material properties of human knee joint cartilage, bone and ligaments to substantiate our critical review of recent advances and current limitations, whilst also serving as a resource for future research in this important area. The critical aspect of our review focuses on the question ‘how systematic or holistic is the material property data that exists for the human knee in terms of its ability to represent a specific human cohort or demographic?’ To evaluate this question we focus on young healthy representation of material properties to understand the current baseline for accurate comparison to old OA representation.

## Survey Methodology

Firstly, published scientific papers were sourced for review that contained material property data of soft and hard tissue from the human knee joint only. The selection criteria are outlined below. Literature search engines were used, including ScienceDirect, PubMed (NCBI), MedLine, SpringerLink and Wiley Online Library. Terminology including *cartilage, bone, ligament, human, knee, joint, femoral, femur, tibia, tibial, anterior, posterior, cruciate, medial, lateral, collateral, material properties, elastic modulus, Young’s modulus, compression, tensile, indentation, FE, model, modelling, three dimensional,* and *computational* were used. All relevant studies meeting search criteria were included in this review.

For cartilage and bone material properties the research must have been on distal femoral and proximal tibia only (excluding patella samples). Studies must have also incorporated the use of compression or indentation techniques for ease of comparison of testing techniques and data obtained (as opposed to tensile elongation, three-point bending, four-point bending or buckling techniques) to collate the elastic modulus, shear modulus or comparable parameters. For ligament material properties studies must have incorporated at least one of the following: anterior cruciate ligament (ACL), posterior cruciate ligament (PCL), medial collateral ligament (MCL) and lateral collateral ligament (LCL) from the human knee tested using tensile techniques. Compression and tensile testing techniques were specifically chosen to mimic primary biological in vivo mechanics. Combined experimental-modelling is sometimes utilised to predict material properties (inverse calculation of material properties from known geometries, loads and deformations) (Robinson et al., 2016); however, this review focuses on more direct measurements of material properties.

Secondly, published scientific papers were sourced for review if they incorporated a 3D FE model of a whole human knee joint. This included any study modelling the femoral and tibial bone and cartilage structures and the four main ligaments of the knee joint—ACL, PCL, MCL and LCL. Studies did not need to include the patella or menisci, as these are less commonly modelled and represented, although were not specifically excluded. Studies not including all these structures were excluded. Studies of meniscectomies, insoles or footwear, joint replacement or arthroplasty mechanics, and ligament reconstructions were also excluded. In addition, we included models representing OA.

Structure, composition and material property data obtained from human tibiofemoral joints were to initially be reviewed separately for cartilage, bone and ligament tissue (Section A—Material Properties), followed by a review of use of data within currently published human tibiofemoral joint FE models (Section B: FE Modelling).

## Section A—Material Properties

### Articular cartilage

Articular cartilage is a type of fibrous connective tissue composed of cells forming between 2% and 15% of the total weight and an extracellular matrix (ECM) forming the remaining 85–98%, of which 65–80% is water (Martini, 1998). Its primary function is to maintain a smooth surface allowing lubricated, near-frictionless movement and to help transmit articular forces, thereby minimising stress concentrations across the joint. It is most commonly found within synovial and diarthrodial joints forming a 1–6 mm thickness and covering the epiphysis of bone. The knee joint is composed of both hyaline and fibrocartilage in the form of articular cartilage covering the end of bones articulating within the joint and fibrocartilage forming the menisci (Martini, 1998).

Material properties of articular cartilage have been widely reported giving compressive, tensile and shear forces at the macro- (Armstrong & Mow, 1982; Setton, Elliott & Mow, 1999; Kleemann et al., 2005), micro- (Stolz et al., 2009; Desrochers, Amrein & Matyas, 2010) and nano-scale (Stolz et al., 2009) within the ECM of multiple species. Various techniques have been utilised including confined and unconfined compression (Kleemann et al., 2005; Hori & Mockros, 1976; Franz et al., 2001) and more recently atomic force microscopy (AFM) (Wen et al., 2012; Wilusz, Zauscher & Guilak, 2013; Wang et al., 2013) and nanoindentation (Taffetani et al., 2014). Custom made indentation instruments have also previously been used to measure articular cartilage stiffness during compression (Hori & Mockros, 1976; Kempson, Freeman & Swanson, 1971; Lyyra et al., 1995; Kiviranta et al., 2008) as well as being used to calculate dynamic modulus (Kiviranta et al., 2008), creep modulus (Kempson, Freeman & Swanson, 1971), shear, bulk and elastic modulus and Poisson’s ratio (Hori & Mockros, 1976).

One of the first studies to explore human knee joint cartilage material properties utilised uniaxial confined compression on 20 proximal tibia samples. Age and gender of donors were not specified; however each sample was classified with a grade of OA using the Bollet system (Bollet, Handy & Sturgill, 1963 cited in Hori & Mockros (1976)). Progressive compression loads were manually applied giving an elastic modulus between 1.3 and 10.2 MPa. When categorising elastic modulus to grade of OA averages were 6.82, 6.74, 4.76 and 2.99 MPa for grades 0, 1, 2 and 3 respectively, although this correlation was not significant (Hori & Mockros, 1976). Testing specifications and resultant data can be seen in Table 1 alongside information from all reviewed human knee joint cartilage material property research.

**Table 1:** Summary of cartilage material properties.

Author	Quantity and locality	Age, gender and health status	Testing technique	Results per Cohort: elastic modulus (MPa)	
Hori & Mockros (1976)	20 × Donors	Age: NS	Uniaxial confined compression 10–30.4 mm indenter	*Healthy and OA grade 1*	1.3–10.2
	Proximal tibia	Gender: NS			
		Health: healthy and OA grade 1			
Shepherd & Seedhom (1997)	5 × Donors	Age: NS	Spring-loaded indentation 1.59 mm indenter	*Healthy*	2.6–18.6
	Femoral condyle and tibial plateau	Gender: NS			
		Health: healthy			
Shepherd & Seedhom (1999a)	11 × Donors	Age: 33–80	Spring-loaded indentation 1.59 indenter	*Healthy*	6.0–11.8
	Femoral condyle and tibial plateau	Gender: 8F/3M;			
		Health: healthy			
Franz et al. (2001)	24 × Femoral	Age: 32–89	Handheld indentation 1.0 mm indenter	*Healthy and OA grade 1*	4.3–4.9
	Condyle	Gender: NS			
		Health: healthy and OA grade 1			
Kleemann et al. (2005)	21 × Donors	Age: 70 ± 13	Uniaxial unconfined compression	*OA grade 1*	0.5
	Tibial plateau	Gender: 15 F/6 M;		*OA grade 2*	0.4
		Health: OA grades 1–3		*OA grade 3*	0.3
Thambyah, Nather & Goh (2006)	7 × Donors	Age: 62–70	Uniaxial unconfined compression 1.0 mm indenter	*Healthy*	2.1–5.1
	Tibia	Gender: M			
		Health: healthy			
Wen et al. (2012)	3 × Donors	Age: 35–59	AFM 10 nm indenter	*Healthy OA grade 1*	2650.0–3700.0* 2370.0–5640.0*
	Knee samples	Gender: F			
		Health: healthy and OA grade 1			
Wilusz, Zauscher & Guilak (2013)	8 × Donors	Age: 53–83	AFM	*Healthy*	0.1 and 0.3
	Femoral condyle	Gender: NS	5 μm indenter	*PCM and ECM*	0.1 and 0.5
		Health: healthy and OA grades 2–3		*OA grade 2–3 PCM and ECM*	
Wang et al. (2013)	5 × Donors	Age: NS	AFM	*Healthy*	0.2
	Femoral condyle	Gender: NS	40 nm indenter	*OA grade 1*	0.6
		Health: healthy and OA grade 1–3		*OA grade 2–3*	0.2

**Notes:**

Summary of current literature for human knee cartilage material property compression or indentation testing including age, gender, health status of specimens, number and location of samples tested and technique used to obtain elastic modulus values.

NS, not specified; F, female; M, male; OA, osteoarthritis; AFM, atomic force microscopy; ECM, extra cellular matrix; PCM, peri-cellular matrix.

* Samples were dehydrated prior to testing.

In more recent decades there has been considerable focus on microscale unconfined compression testing. In consecutive studies by Shepherd & Seedhom (1997, 1999a), human femoral condyle and tibial plateau cartilage were tested. Earlier research utilised a total of five donors although no age or gender was specified. Results indicated an elastic modulus of between 2.6 and 18.6 MPa depending on physiological loading rate (Shepherd & Seedhom, 1997). In the latter study 11 humans cadavers (three males and eight females, aged 33–80 years old) were tested giving an elastic modulus of 6.0–11.8 MPa (Table 1) across all cadavers with no correlation to age (Shepherd & Seedhom, 1999a).

Thambyah, Nather & Goh (2006) tested cartilage from seven fresh frozen healthy human male tibias (62–70 years old) using uniaxial tensile testing at a rate of 300 kPa/s to compare articular cartilage from beneath the menisci to that independent from the menisci. Results showed an individual mean elastic modulus from all seven cadavers between 2.13 and 5.13 MPa (Table 1) across varying testing locations. Hydration maintenance was not specified within the methodology.

**Figure 1:**
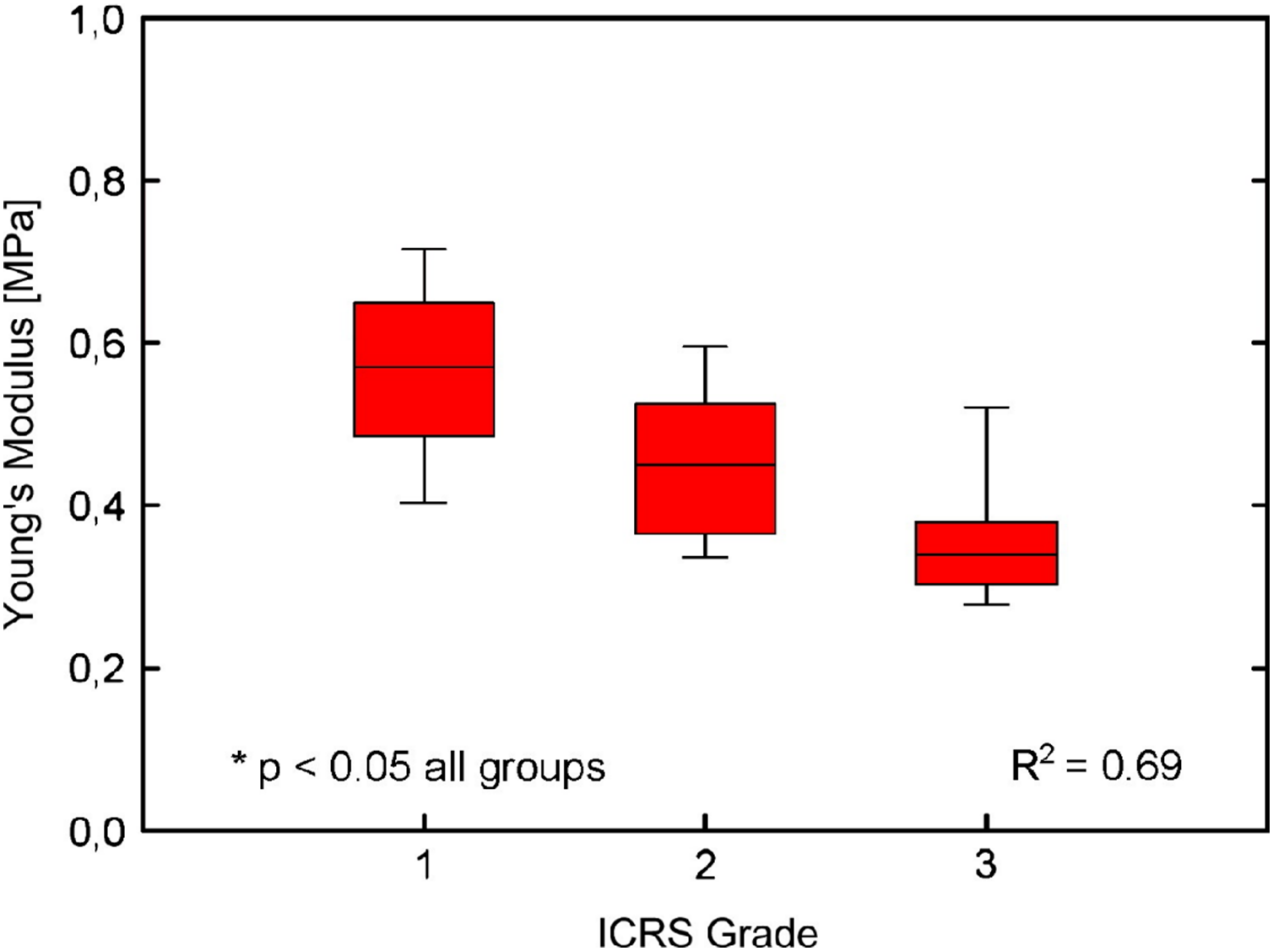
Cartilage stiffness during degeneration. Stiffness reduction of degenerated cartilage with increasing International Cartilage Repair Society (ICRS) Grade related to boxplots displaying median values and interquartile range. (Adapted from Kleemann et al. (2005): Elsevier License Permission: 4226450501899).

Kleemann et al. (2005) explored the macroscopic composition of articular cartilage within 15 females and 6 males OA tibial plateau samples (70 ± 13 years old). Research obtained architectural data from histology using haematoxylin and eosin staining and elastic modulus of cartilage was determined by unconfined uniaxial compression. An inverse correlation was observed between the elastic modulus of the articular cartilage against the International Cartilage Repair Society (ICRS) grade (Brittberg & Peterson, 1998) seen in Fig. 1 (Grade 1 0.50 MPa, Grade 2 0.37 MPa, and Grade 3 0.28 MPa (Table 1)). The research also suggested a relationship between changes in histology, structure and mechanics of the articular cartilage during all stages of OA degeneration although this was not compared with age of donor. Moreover Bae et al. (2003) found decreased indentation stiffness and an increased ICRS score was associated with degeneration of cartilage rather than with age or cartilage thickness. This suggests that it is possible to reliably distinguish degeneration of cartilage by microscopic histological analysis and macroscopic observations.

Franz et al. (2001) used a handheld indenter with a constant load of 300 μm to collate the shear modulus of 24 human cartilage samples (32–89 years old) obtained from the medial and lateral femoral condyles. Shear modulus was converted to elastic modulus (using the Poisson’s ratio expressed in the original research) for the purpose of this paper, which were 4.32 MPa and 4.88 MPa (Table 1) in the lateral and medial femoral condyles respectively; however this was not correlated to the age of cadaver. Cartilage samples were graded for OA using the Mankin system (Mankin et al., 1971) and results indicated a positive correlation between a slightly roughened cartilage surface and stiffness at the medial femoral condyle. However, it should be noted that no samples presented with gross fibrillation or surface irregularities. Sample shear modulus was, however, presented in age categories with corresponding proteoglycan and collagen content which are known to adapt during ageing and disease.

The development of increasingly sophisticated testing techniques has further advanced our understanding of cartilage material properties by allowing measurements to be made at the nanoscale. With the use of nanoscale indentation stiffening of cartilage due to age-related influences alongside stiffness differences in healthy and OA cartilage can be detected more accurately in comparison to microindentation (Stolz et al., 2009). It has been shown that microindentation is either unable to detect such changes or produces a lower stiffness measurement when compared to nanoindentation leading some to question its accuracy (Stolz et al., 2004, 2009). Additionally, stiffness is higher in articular cartilage collagen fibrils than in proteoglycans; however when measured at microscale, this differentiation may not be detected (Loparic et al., 2010). A change in the structure and content of proteoglycans often accompanies the process of OA along with reduced stiffness through loosening of the collagen network causing alteration to the material properties, further enhancing the need for testing at the nanoscale (Wang et al., 2013).

Incorporating nanotechnology, Wen et al. (2012) utilised AFM at a loading rate of 2.11 nm/s to test elastic modulus of tibial plateau articular cartilage fragments obtained from three female patients undergoing arthroplasty surgery. Samples from the surface, superficial middle, deep middle and bone–cartilage interface regions were graded for OA with the Outerbridge scoring system (Outerbridge, 1961). Collagen fibres were obtained from the overlap zone from each layer which can be mechanically stiffer than collagen fibres in the gap region (Minary-Jolandan & Yu, 2009). Results show there is a significant mechanical stiffening of individual human collagen fibrils between healthy (aged 35 years old) and mild OA (aged 52 and 59 years old), at the surface of articular cartilage (2,650–3,110 MPa respectively) through to the bone–cartilage interface (3,700–5,640 MPa respectively) (Table 1). It must be noted that tissue samples were dehydrated with ethanol prior to testing which will alter the true mechanical properties of cartilage; however the aim of this research was to identify the differences in elastic modulus of healthy and OA tissues where mechanical alterations would change simultaneously in both healthy and OA samples.

Wilusz, Zauscher & Guilak (2013) also used AFM at a rate of 15 μm/s on eight human femoral condyles (six females and two males) aged 53–83 years old. Cadavers were graded for OA using the Collins System (Collins, 1939, 1949 cited in Wilusz, Zauscher & Guilak (2013)) giving four healthy and four OA samples grades 2–3. Results indicate that elastic modulus of the pericellular matrix (PCM) decreased in OA samples (0.096 ± 0.016 MPa) when compared to healthy controls (0.137 ± 0.022 MPa). Also the ECM elastic modulus was decreased in OA samples (0.270 ± 0.076 MPa) when compared to healthy controls (0.491 ± 0.112 MPa) (Table 1); although this was only significant on the medial femoral condyle. In agreement, Wang & Peng (2015) used AFM to quantify elastic modulus of 12 knee articular cartilage samples (age and gender not specified) in various grades of OA and found an increase in elastic modulus in the presence of mild and moderate OA but a decrease with severe OA, although actual values are not stated.

Atomic force microscopy has also been used to identify nanoscale adaptations at varying indentation depths in five human (age and gender not specified) femoral condyles obtained from healthy, mild and severe OA cartilage (Wang et al., 2013). Cartilage samples were graded using the Outerbridge scoring system (Outerbridge, 1961) and exposed to PBS during testing to maintain hydration. Stiffness was higher at a lower indentation depth for all cohorts; however, stiffness was highest with mild OA (0.61 MPa) and lowest with healthy controls (0.16 MPa) when comparing to severe OA (0.19 MPa) (Table 1) (Wang & Peng, 2015).

### Bone

There are two different types of bone including cortical and trabecular material. The cortical material is found on the outside of bone and is highly dense in nature and the trabecular material is located inside of the bone and has a greater porosity. The low and high densities work in coordination to absorb stresses through the rigid outer surface and strains through the spongy inner material in order to resist breaking or deformation (Nigg & Herzog, 2006; Martini, 1998).

Recent research has started to direct focus onto the relationship between cartilage and bone in the progression of OA. Research has observed abnormal remodelling of subchondral bone in OA showing the trabecular structure alters in density, quantity and separation, with the greatest proliferation in volume evident at the bone–cartilage interface (Kamibayashi et al., 1995; Bobinac et al., 2003). This suggests a synergistic relationship between bone and cartilage during the progression of OA. The role of subchondral bone in OA appears to be an essential component in the initiation and advancement of the disease (Burr, 1998; Lajeunesse & Reboul, 2003; Madry, van Dijk & Mueller-Gerbl, 2010). However research is unclear as to whether disruption of subchondral bone remodelling occurs pre- or post-initiation of OA (Intema et al., 2010; Kuroki, Cook & Cook, 2011). Kuroki, Cook & Cook (2011) suggested that a more comprehensive understanding of the disease mechanisms of OA including material properties of all tissues involved could yield considerable progression in clinical practice and treatment methods.

In previous decades uniaxial compression testing of human femoral and tibial trabecular bone was carried out by several researchers in order to obtain macroscale material properties. Behrens, Walker & Shoji (1974) tested both femoral condyle and tibial plateau trabecular bone samples from six females and four males (40–92 years old) resulting in an elastic modulus of 158.9–277.5 MPa for femoral bone and 139.3–231.4 MPa for tibial samples (Table 2). Testing only femoral condyle trabecular bone, Ducheyne et al. (1977) found a slightly lower elastic modulus of 1.9–166.1 MPa (Table 2) based on donors aged 43–77 years old (four males, two females).

**Table 2:** Summary of bone material properties.

Author	Quantity and locality	Age, gender and health status	Testing technique	Results per Cohort: elastic modulus (MPa)	
Behrens, Walker & Shoji (1974)	10 × Donors	Age: 40–92	Uniaxial compression	*Femoral condyle*	158.9–277.5
	Femoral condyle and tibial plateau trabecular bone	Gender: 6F/4M		*Tibial plateau*	139.3–231.4
		Health: healthy			
Lindahl (1976)	8 × Donors	Age: 14–89	Uniaxial compression	*Males*	34.6
	Tibial plateau trabecular bone	Gender: 4F/4M		*Females*	23.1
		Health: healthy			
Carter & Hayes (1977)	100 × Samples	Age: NS	Uniaxial compression		56.6–83.7
	Tibial plateau trabecular bone	Gender: NS			
		Health: Healthy			
Ducheyne et al. (1977)	6 × Donors	Age: 43–77	Uniaxial compression		1.9–166.1
	Femoral condyle trabecular bone	Gender: 2F/2M			
		Health: healthy			
Goldstein et al. (1983)	5 × Donors	Age: 50–70	Uniaxial compression		4.2–430
	Tibial plateau trabecular bone	Gender: 2F/3M			
		Health: healthy			
Hvid & Hansen (1985)	12 × Donors	Age: 26–83	Uniaxial compression 2.5 mm indenter	*Medial*	13.8–116.4
	Tibial plateau trabecular bone	Gender: 3F/9M		*Lateral*	9.1–47.5
		Health: healthy			
Zysset, Sonny & Hayes (1994)	6 × Donors	Age: 61–91	Uniaxial compression	*Subchondral epiphyseal/metaphyseal*	31.0–1116.0*
	Tibial trabecular bone	Gender: NS			8.0–1726.0*
		Health: OA grades 1–3			
Rho, Tsui & Pharr (1997)	2 × Donors	Age: 57 and 61	Nanoindentation 20 nm indenter		22500.0–25800.0
	Tibial cortical bone	Gender: M			
		Health: healthy			
Burgers et al. (2008)	10 × Donors	Age: 45–92	Uniaxial compression		131.0–664.0
	Femoral condyle trabecular bone	Gender: NS			
		Health: healthy			

**Notes:**

Summary of current literature for human knee bone material property compression or indentation testing including age, gender, health status of specimens, number and location of samples tested and technique used to obtain elastic modulus values.

GNS, gender not specified; F, female; M, male; OA, osteoarthritis.

* Elastic modulus value for individual OA grade not specified—value taken as approximation from graph.

Carter & Hayes (1977) tested 100 human trabecular bone samples (age and gender unspecified) from tibial plateaus by uniaxial compression and found an elastic modulus between 56.6 and 83.7 MPa (Table 2). Also using uniaxial compression, Lindahl (1976) tested four females and four males human cadavers (14–89 years old) showing a higher elastic modulus in males (average 34.6 MPa) compared to females (average 23.1 MPa) (Table 2).

Interestingly, as well as differences between male and female cadavers, material properties also vary according to anatomical location. Goldstein et al. (1983) utilised uniaxial compression testing to determine the elastic modulus of trabecular bone from the tibial plateau from five cadavers (50–70 years old) across varying depths of the joint. Results showed high variation across cadavers and testing location (4.2–430 MPa (Table 2)) with the highest values at load bearing sites. Utilising an alternative method, Hvid & Hansen (1985), used an osteopenetrometer on the tibial plateau of 12 healthy human donors aged 26–83 years old (three females and nine males). Medial tibial plateau samples had an elastic modulus of 13.8–116.4 MPa and lateral tibial plateau samples had a lower elastic modulus of 9.1–47.5 MPa (Table 2) further evidencing high variability in material properties across the joint.

Burgers et al. (2008) obtained four male and four female human cadavers (totalling 10 femurs aged 45–92 years old). Cylindrical trabecular specimens (*n* = 28) were tested using unconfined compression. Results were separated into superior or inferior and medial or lateral samples giving a pooled elastic modulus of 376 ± 347 MPa (Table 2) with the greatest variation apparent between superior and inferior femoral condyle samples.

**Figure 2:**
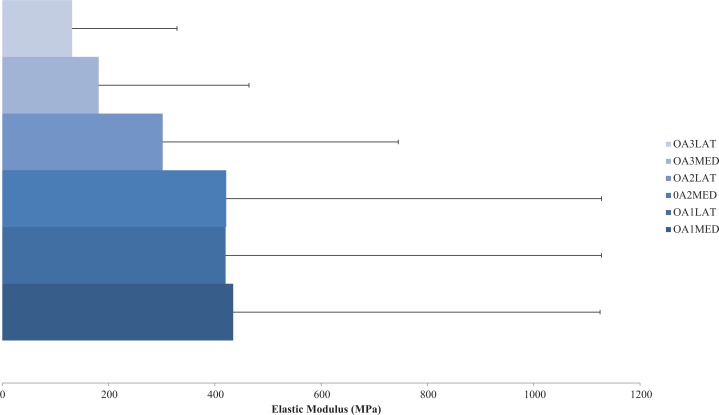
Compressive elastic modulus of subchondral bone in osteoarthritis. Compressive axial elastic modulus of subchondral bone for a range of osteoarthritis (OA) grades (1–3). Average elastic modulus decreases with degenerative grade in the medial (MED) and especially lateral (LAT) compartments. (Redrawn from Zysset, Sonny & Hayes (1994): Elsevier License Permission: 4226540285665).

Previous studies researching human knee bone material properties, specifically in OA, are abundantly missing; however one study by Zysset, Sonny & Hayes (1994) explored human tibial material properties from six cadavers (61–91 years old) with grades 1–3 OA, scored using the Ahlback system (Ahlback, 1968). Compression tests were conducted on cuboidal specimens giving an axial elastic modulus of the subchondral trabecular bone between 31 and 1,116 MPa which decreased with increasing grades of OA. Although epiphyseal and metaphyseal trabecular bone samples showed that elastic modulus increased with OA grade in the axial (102–1,726 MPa) and coronal (8–287 MPa) planes (Table 2). Corresponding OA grade and elastic modulus values can be seen in Fig. 2.

In more recent years, testing bone at the tissue level has proven to be more accurate (Nigg & Herzog, 2006) particularly for the inclusion of FE models; however this has rarely been applied to femoral or tibial human bone. Using nanoindentation Rho, Tsui & Pharr (1997) explored the tissue level material properties of a single osteon and interstitial lamellae of two longitudinal human (57 and 61 years old) tibial cortical bone. Results presented an elastic modulus of 22,500 MPa and 25,800 MPa for osteon and interstitial lamellae samples respectively (Table 2).

### Ligaments

Ligaments are soft tissues that are fibrous in nature and composed primarily of collagen. They have a hierarchal structure of fibres, fibrils, subfibrils, microfibrils and tropocollagen but also contain water, proteoglycans and several glycoproteins. They function to guide and resist motion at a joint by connecting bone to bone. It has also been suggested that they act as a strain sensor to restrict degrees of freedom in order to stabilise the joint and prevent excessive movement (Harner et al., 1995; Woo et al., 2006). Ligaments have direct and indirect insertions into the bone and periosteum respectively allowing variation in fibre bundles to respond to different movements and resist loading during ranges of rotation at the joint. The entheses portion of the ligament is stiffer compared to the medial portion allowing decreased concentrations of stress and therefore reducing the opportunity for damage or tears at the bone–ligament interface (Woo et al., 2006).

**Figure 3:**
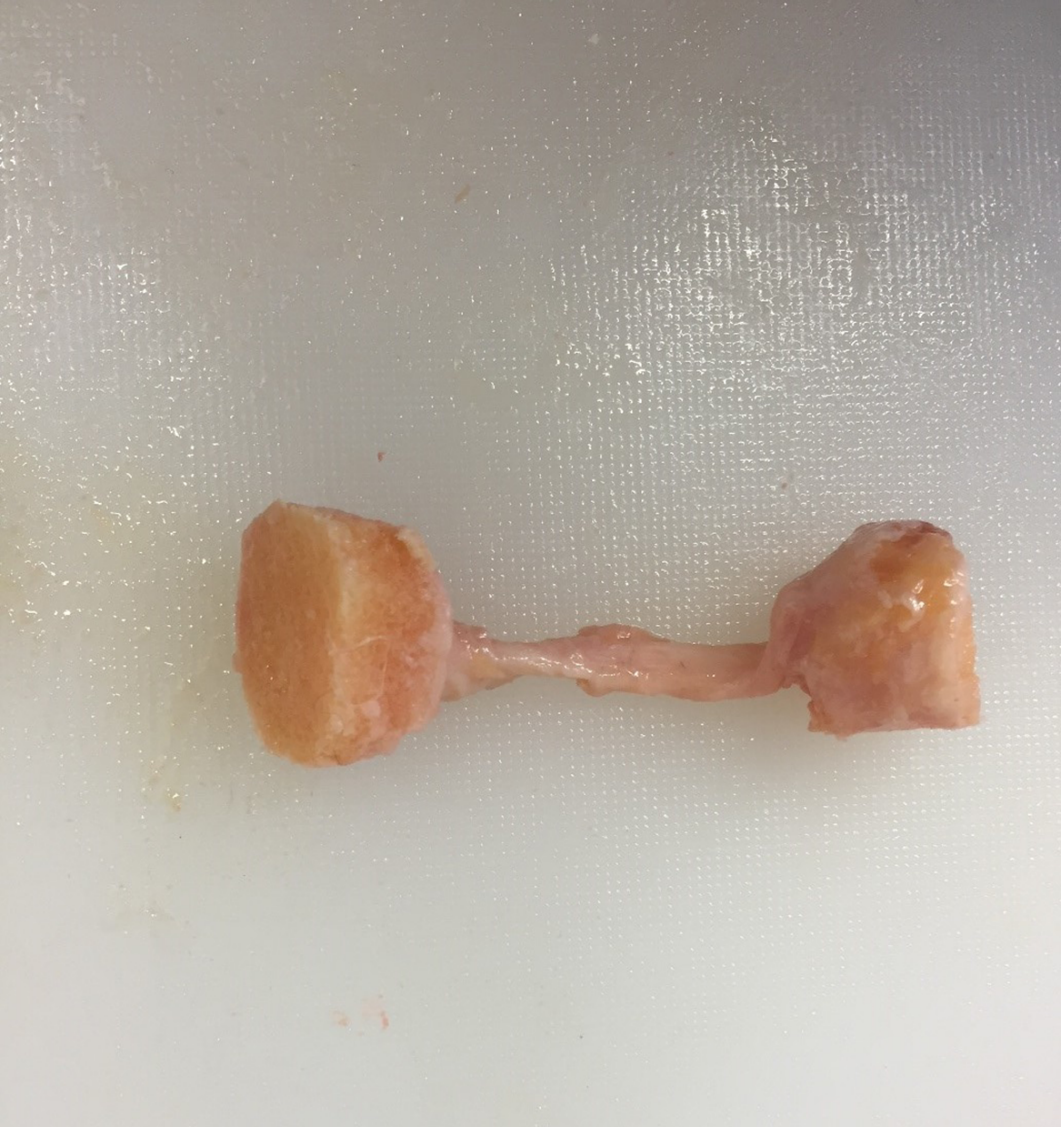
Example bone–ligament–bone sample. Photograph of a medial collateral bone–ligament–bone sample. Image from the authors’ own work. (Ethics granted by NRES (15/NS/0053)).

When measuring material properties of knee ligaments (ACL, PCL, MCL and LCL) typical analyses includes tensile stress and strain at ultimate failure, tangent modulus and strain energy density, primarily obtained using a tensile testing machine. These parameters are tested in vitro by taking either a cross-section of the involved ligament (Quapp & Weiss, 1998) or more commonly a bone–ligament–bone sample (e.g. Fig. 3). During this process bone blocks are ordinarily embedded within polymethyl-methacrylate (PMMA) and the ligaments are wrapped in saline soaked gauze for protection (Harner et al., 1995; Butler et al., 1992; Momersteeg et al., 1995; Hewitt et al., 2001; Robinson, Bull & Amis, 2005; Bonner et al., 2015). Additionally samples may be tested as a whole structure or divided into anatomical fibre bundles. Woo et al. (2006) suggests that the ACL has an anteromedial and posterolateral bundle and the PCL has an anterolateral and posteromedial bundle which are loaded differently. Ligaments therefore may need to be separated during tensile testing, in order to gain a true understanding of their unique material properties. A summary of the reviewed ligament material property research papers is provided in Table 3.

**Table 3:** Summary of ligament material properties.

Author	Quantity and locality	Age, gender and health status	Testing technique	Results					
				*Tissue type*	Stiffness (N/mm)	Failure load (N)	Elastic modulus (MPa)	Max stress (MPa)	Max strain (%)
Trent, Walker & Wolf (1976)	7 × ACL, PCL, MCL and LCL	Age: 29–55	Bone–ligament–bone	*ACL*	138.3	620.8			
		Gender: NS		*PCL*	179.5	658.0			
		Health: healthy		*MCL*	70.6	515.8			
				*LCL*	59.8	376.6			
Noyes & Grood (1976)	26 × ACL	Age: 16–86	Bone–ligament–bone	*Young*	182.0	1730.0	*111.0*	*37.8*	*44.3*
		Gender: NS		*Old*	129.0	734.0	*65.3*	*13.3*	*30.0*
		Health: healthy							
Butler, Kay & Stouffer (1986)	3 × ACL, PCL and LCL	Age: 21–30	Bone–ligament–bone	*ACL*			278.0–310.0*	30.0–40.0*	14.0–16.0*
		Gender: 2F/1M		*PCL*			280.0–447.0*	34.0–44.0*	14.0–19.0*
		Health: healthy		*LCL*			375.0–25.0*	31.0–43.0*	11.0–17.0*
Woo et al. (1991)	27 × ACL bilateral	Age: 22–97	Bone–ligament–bone	*Young 22–35*	218.0–242.0	1602.0–2160.0			
		Gender: NS		*Middle 40–50*	192.0–220.0	1160.0–1503.0			
		Health: healthy		*Old 60–97*	124.0–180.0	495.0–658.0			
Butler et al. (1992)	7 × ACL	Age: 26 ± 4	Bone–ligament–bone	*Anteromedial fibers*			238.1	54.7	19.1
		Gender: NS		*Anterolateral fibers*			285.9	30.6	16.1
		Health: healthy		*Posterior fibers*			154.9	15.4	15.2
Race & Amis (1994)	10 × PCL	Age: 53–98	Bone–ligament–bone	*Anterolateral fibers*	*347.0*	1620.0	248.0	35.9	18.0
		Gender: NS		*Posteromedial fibers*	*77.0*	258.0	145.0	24.4	19.5
		Health: healthy							
Harner et al. (1995)	5 × PCL	Age: 48–77	Bone–ligament–bone	*Anterolateral fibers*	120.0	1120.0			
		Gender: NS		*Posteromedial fibers*	57.0	419.0			
		Health: healthy							
Quapp & Weiss (1998)	10 × MCL	Age: 62 ± 18	Ligament sample only	*Longitudinal*			38.6		17.1
		Gender: NS		*Transverse*			1.7		1.7
		Health: healthy							
Robinson, Bull & Amis (2005)	8 × MCL	Age: 77 ± 5.3	Bone–ligament–bone	*Superficial MCL*		534.0			
		Gender: NS		*Deep MCL*		194.0			
		Health: healthy		*Posteromedial capsule*		425.0			
Chandrashekar et al. (2006)	17 × ACL	Age: 17–50	Bone–ligament–bone	*ACL total*	250.0	1526.0	113.0	24.4	
		Gender: 9F/8M		*Male*	308.0	1818.0	128.0	26.4	
		Health: healthy		*Female*	199.0	1266.0	99.0	22.8	

**Notes**:

Summary of current literature for human knee ligament material properties including location and number of samples, age, gender, health status of donors, testing technique and resultant data. N.B. for comparison purposes only those papers testing ligaments to failure will be included in this table.

GNS, gender not specified; F, female; M, male; ACL, anterior cruciate ligament; PCL, posterior cruciate ligament; MCL, medial collateral ligament; LCL, lateral collateral ligament.

* Values are approximated from graph data.

Harvesting a cross-sectional area of a ligament, Quapp & Weiss (1998) explored the longitudinal and transverse mechanical behaviour of the MCL from 10 human cadavers (62 ± 18 years old). Specimens were preconditioned and loaded to failure. Results included average tensile strength (38.6 and 1.7 MPa), average ultimate strain (17.1% and 1.7%) and average tangent modulus (332.2 and 11.0 MPa) for longitudinal and transverse specimens respectively (Table 3).

Further research on the tensile properties of ligaments utilised the bone–ligament–bone method. One of the first studies to explore ligament material properties harvested the ACL, PCL, MCL and LCL from seven healthy human cadavers aged 29–55 years old (gender not specified). Ligaments were preconditioned over five cycles and loaded to failure at 100% strain rate, which is a change in strain equivalent to the initial length of the ligament. Stiffness was measured at 138.3, 179.5, 70.3 and 59.8 N/mm for the ACL, PCL, MCL and LCL respectively, whilst failure load resided at 620.8, 658.0, 515.8 and 376.6 N (Table 3) (Trent, Walker & Wolf, 1976).

Noyes & Grood (1976) tested young (16–26 years old) and old (48–86 years old) anterior cruciate bone–ligament–bone material properties, also at a 100% strain rate, although excluded any preconditioning. The research found a reduction in stiffness (129 and 182 N/mm), failure load (734.0 and 1730.0 N), elastic modulus (65.3 and 111.0 MPa), maximum stress (13.3 and 37.8 MPa) and strain (30.0% and 44.3%) when comparing older samples to younger samples respectively (Table 3).

Butler, Kay & Stouffer (1986) also tested young (21–30 years old) ACL, PCL and LCL elastic modulus (278–447 MPa), maximum stress (30–44 MPa) and maximum strain (11–19%) where ranges were inclusive of all ligaments. Approximate values are given in Table 3 estimated from presented graphs (Butler, Kay & Stouffer, 1986). The ligaments were divided into their fibre bundles and tested to failure at a 100%/s strain rate (Table 3). Further research by Butler et al. (1992) looked at the differences in seven human ACL (26 ± 4 years old) divided into anteromedial, anterolateral and posterior fibre bundles. Specimens were not exposed to preconditioning but were loaded to failure at a 100%/s strain rate. This resulted in anterior fibres having a higher maximum modulus (284 MPa), stress (38 MPa) and strain rate (17.6%) when compared to posterior fibres (155 MPa, 15 MPa, 15.2%) at failure (Table 3).

Race & Amis (1994) and Harner et al. (1995) loaded to failure the anterolateral and posteromedial fibres bundles of the human PCL. Race & Amis (1994) obtained 10 samples from donors aged 53–98 years old which resulted in higher stiffness (347.0 and 770 N/mm), failure load (1620.0 and 258.0 N), elastic modulus (248.0 and 145.0 MPa) and maximum stress (35.9 and 24.4 MPa) for the anterolateral fibres in comparison to the posteromedial fibres respectively (Table 3). Interestingly maximum strain was lower for the anterolateral fibres (18.0%) when compared to the posteromedial fibres (19.0%). Harner et al. (1995) tested five samples (48–77 years old) and also found a higher failure load in the anterolateral fibres (1120.0 N) in comparison to the posteromedial fibres (419.0 N) (Table 3) showing in both studies wide variation depending on the location of the tissue.

A more recent study by Robinson, Bull & Amis (2005) harvested three sections of the femur–MCL–tibia complex from eight humans (77 ± 5.3 years old), namely the superficial MCL (SMCL), deep MCL (DMCL) and posteromedial capsule (PMC) based on fibre orientation and tested samples using the bone–ligament–bone approach. The SMCL is often used to define the overall MCL length; however, it is thought that each section tenses and fully elongates under different loading axis or directions and functions to stabilise the knee joint in various ways. Samples were preconditioned and loaded to failure resulting in failure loads of 534, 194 and 425 N for the SMCL, DMCL and PMC respectively (Table 3). The results indicated a bony avulsion in 75% of tested samples after which the bone was removed and the end of the ligament was attached directly in the clamps and re-loaded to failure. Additionally mid-substance failure of the ligament as opposed to bony avulsion equated to 74% higher maximum load.

**Figure 4:**
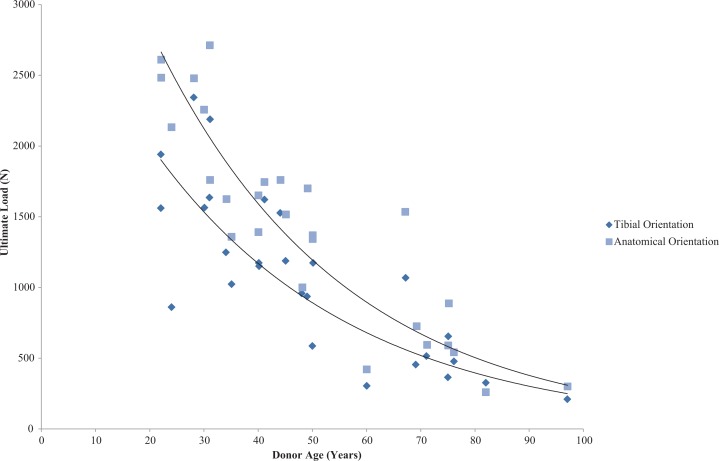
Effect of specimen age on anterior cruciate ligament ultimate load. Effect of specimen age on anterior cruciate ligament (ACL) ultimate load. Data on ultimate load as a function of specimen age and orientation demonstrated that the strength of the ACL decreases in an exponential manner. (Redrawn from Woo et al. (1991): Sage License Permission: 4226541340810).

Further variations in tensile properties can exist due to the angle of the femur in correlation to the tibia and the loading axis in correlation to ligament fibre loading direction. Woo et al. (1991) preconditioned and tested the ACL to failure along both the tibial and ligament axis and found higher stiffness values on the ligament axis with increasing extension angle when testing young and old cadavers. Significant variations in anatomical orientation failure load were apparent between age groups: 2,160 N for 22–35 years old (*N* = 9), 1,503 N for 40–50 years old (*N* = 9) and 658 N for 60–97 years old (*N* = 9) (Table 3) as seen in Fig. 4. However, there was no correlation between age and orientation.

Interestingly, Chandrashekar et al. (2006) found gender-based differences in tensile properties showing human female ACL (*N* = 9) (17–50 years old) had 22.49% lower elastic modulus and 8.3% and 14.3% lower maximum strain and stress respectively when compared to human male ACL (*N* = 8) (26–50 years old) (Table 3). These differences can be partially accounted for due to the physically smaller size of the female ACL (Anderson et al., 2001; Chandrashekar, Slauterbeck & Hashemi, 2005); however, when adjusted for covariates the tensile properties of the ACL are still lower. This may in turn explain the higher rates of ACL injuries in female athletes (Chandrashekar et al., 2006).

Finally an analysis by Momersteeg et al. (1995) chose not to separate the fibre bundles but instead tilted the orientation of the loading axis at 5° increments (up to 25°) to recruit different fibres at varying angles to explore the changes in tensile properties during sub-ultimate testing. Bone–ligament–bone samples were harvested for the ACL, PCL, MCL and LCL of five human cadavers (63–81 years old) and subjected to preconditioning before applying up to 7% and 10% strain rates for the collateral and cruciate ligaments respectively. Results indicate that strain levels were higher for cruciate ligaments than collateral ligaments and for every 5° of tilt there was a decrease in tensile stiffness (averages: −11.6 Nmm^−1^ ACL, −20.96 Nmm^−1^ PCL, −2.66 Nmm^−1^ MCL, −3.76 Nmm^−1^ LCL) (Table 3). The research suggests there is a greater decrease in stiffness for the cruciate ligaments as they have a shorter and wider morphology when compared to the long thin nature of collateral ligaments. These authors go on to conclude that ligaments are highly sensitive to a small change in orientation and therefore unidirectional tensile testing is not effective at defining ligament stiffness properties (Momersteeg et al., 1995).

## Section B: FE Modelling

Freutel et al. (2014) presented a non-systematic review on the current research on FE modelling within soft tissues with a specific focus on the human knee joint and intervertebral disc. They reviewed strategies for modelling various material properties, considering the interaction between soft tissues during contact and their sensitivity to changes in properties and environment (i.e. loading and boundary conditions). Their review concluded that inaccuracy or abstraction in each of these areas could manifest into important limitations in structurally complex models such as those of the human knee joint. Material property definition was cited by Freutel et al. (2014) and indeed by others (Gardiner & Weiss, 2003), as a research area with potential for significant improvement either through improved modelling approaches or in vivo inclusion of material properties particularly given the advances in techniques for characterising biological tissue behaviour in recent decades.

Following on from this review of available material property data for human knee joint tissues in ‘Section A—Material Properties’ (above) we focus subsequently on the material property data that has actually been utilised in published whole-joint FE models of the human knee. It is our hope that clarifying the FE models that currently exist in the literature and their accuracy according to how they have obtained their material property data (i.e. primary data collection or from various data sets and donors) will help identify gaps within the knowledge and aid future directions for research.

Advances in FE modelling have allowed researchers to present cartilage as a non-linear anisotropic material with varying material properties as opposed to the traditional representation of a linear elastic isotropic material. This advance means cartilage can now be represented with greater biofidelity and therefore computational predictions of behaviours are likely to be more accurate. Several authors have adopted this advanced approach in recent years (Tanska, Mononen & Korhonen, 2015; Halonen et al., 2013); however, due to the complexity and computation expense of such models, individual tissues are often modelled in isolation, meaning other structures not relevant to the research hypothesis are excluded. Although useful in particular applications, if representing OA of the knee joint, modelling tissues in isolation has its limitations. It is now well established that this is a disease of the entire joint with molecular crosstalk and changes in subchondral bone structure (Lories & Luyten, 2011; Mahjoub, Berenbaum & Houard, 2012), and histological evidence of ligament structural changes (Mullaji et al., 2008). Therefore if investigating such diseases it is now inherently clear that whole-joint representation is needed to fully understand the implications of tissue interaction and disease progression on the knee joint.

When cartilage is modelled with linear elasticity it assumes an instantaneous response to stress and strain; however, nonlinear representation allows for viscoelastic or time dependent factors such as those represented in Mononen et al. (2011, 2012). It is now well established that cartilage and ligaments are nonlinear and viscoelastic and material property testing is starting to incorporate time-dependent testing by including a hold period. This review is intended to analyse whole-joint representations only. Studies presenting only singular tissues of the human knee joint with more detailed material behaviours are outside the scope of this review, although the recent efforts in modelling hyperelastic formulations of cartilage and efforts towards representing tissue anisotropy and viscoelasticity are summarised below.

Modelling cartilage as a fibril reinforced poroviscoelastic tissue with multiple material properties, Tanska, Mononen & Korhonen (2015) explored chondrocyte compression during walking, whilst research by Halonen et al. (2013) explored cartilage deformation under large compression. Further, work by Dabiri & Li (2013) also modelled cartilage with depth-dependent properties, making it possible to use a fibril-reinforced model to explore inhomogeneity and fluid pressurisation within the tissue. Meng et al. (2014) considered cartilage as a fibril reinforced biphasic material to explore knee joint contact behaviour under body weight. Other examples of research representing cartilage as a poroelastic or poroviscoelastic material include the work of Kazemi et al. (2011) and Mononen et al. (2011, 2012). These studies represented whole-joints and are therefore discussed in more detail below.

For the purpose of this review, research papers that have presented a FE model of a healthy human knee joint incorporating the femur, tibia, cartilage and four major ligaments each within a 3D form will be presented, addressing how and where these models have sourced material property data for their models. Following this, models that have included all these structures but most commonly represented them in a simplified form of one, two and 3D forms will also be reviewed. Finally the existing attempts to simulate the effects of OA within the knee joint using FE models will be discussed.

### 3D FE models of healthy human knee joints

This review reveals that FE models most commonly use previously published data for material properties; however, there is usually a lengthy referencing chain when tracing these material properties to their original and primary data research article. Material properties are likely to vary with age, gender and disease status (Kleemann et al., 2005; Lindahl, 1976; Woo et al., 1991; Chandrashekar et al., 2006) and therefore donor demographics in previously published material property studies will undoubtedly impact upon the quantitative results obtained in FE analyses. Our review highlights a wide spectrum of matches in this respect to the extent that the absence of appropriate data has in some cases led to the use of non-human material properties in FE models of the knee. Material property sources from reviewed FE models are summarised in Table 4.

**Table 4:** Summary of human knee finite element models.

	Purpose	Bone	Cartilage	Menisci	Ligaments
Blankevoort et al. (1991)	Rigid and deformable articular contact during axial and varus/valgus rotations	N/a	Information untraceable**	N/a	Human (ACL, PCL, LCL) 43–74 years
					Some information untraceable (Butler, Kay & Stouffer, 1986; Blankevoort, Huiskes & De Lange, 1988***)
Blankevoort & Huiskes (1991)	Ligament–bone interaction during axial and varus/valgus rotations	N/a	Information untraceable**	N/a	Human (ACL, PCL, LCL) 43–74 years
					Some information untraceable (Butler, Kay & Stouffer, 1986; Blankevoort, Huiskes & De Lange, 1988***)
Bendjaballah, Shirazi-Adl & Zukor (1995)	Articular cartilage deformation under compression up to 1,000 N	N/a	Human (tibial plateau) 48–70 years (Hayes & Mockros, 1971)	Human (menisci) 29–45 years	Human (ACL, PCL, LCL) 53–98 year* (Butler, Kay & Stouffer, 1986; Race & Amis, 1994)
				Some information untraceable (Tissakht & Ahmed, 1995)	
Bendjaballah, Shirazi-Adl & Zukor (1997)	Role of collateral ligaments in varus–valgus motion	N/a	Human (tibial plateau) 48–70 years (Hayes & Mockros, 1971)	Human (menisci) 29–45 years	Human (ACL, PCL, LCL) 53–98 year* (Butler, Kay & Stouffer, 1986; Race & Amis, 1994)
				Some information untraceable (Tissakht & Ahmed, 1995)	
Jilani, Shirazi-Adl & Bendjaballah (1997)	Non-linear elastostatic response of ligaments during axial rotation with 10 N torque	N/a	Human (tibial plateau) 48–70 years (Hayes & Mockros, 1971)	Human (menisci) 29–45 years	Human (ACL, PCL, LCL) 53–98 year* (Butler, Kay & Stouffer, 1986; Race & Amis, 1994)
				Some information untraceable (Tissakht & Ahmed, 1995)	
Bendjaballah, Shirazi-Adl & Zukor (1998)	Anterior–posterior drawer forces on cartilage under compression up to 400 N loads	N/a	Human (tibial plateau) 48–70 years (Hayes & Mockros, 1971)	Human (menisci) 29–45 years	Human (ACL, PCL, LCL) 53–98 year* (Butler, Kay & Stouffer, 1986; Race & Amis, 1994)
				Some information untraceable (Tissakht & Ahmed, 1995)	
Li et al. (1999)	Ligament forces in response to internal–external moments up to 10 Nm	N/a	Information untraceable**	N/a	Human (ACL, PCL, LCL) 43–74 years
					Some information untraceable (Butler, Kay & Stouffer, 1986; Blankevoort, Huiskes & De Lange, 1988***)
Li, Lopez & Rubash (2001)	Cartilage contact stress sensitivity analysis with compression up to 1,400 N	N/a	Information untraceable	N/a	Human (ACL, PCL, LCL) 43–74 years
					Some information untraceable (Butler, Kay & Stouffer, 1986; Blankevoort, Huiskes & De Lange, 1988***)
Moglo & Shirazi-Adl (2003)	Cruciate ligament behaviour under 100 N femoral load in flexion	N/a	Human (tibial plateau) 48–70 years (Hayes & Mockros, 1971)	Human (menisci) 29–45 years	Human (ACL, PCL, LCL) 53–98 year* (Butler, Kay & Stouffer, 1986; Race & Amis, 1994)
				Some information untraceable (Tissakht & Ahmed, 1995)	
Beillas et al. (2004)	In vivo kinematics and ground reaction forces during one leg hop with compression up to 1,790 N	Human (proximal femur and mid femur) 28–91 years*	Human (tibial plateau) age not specified*	Human (menisci) age not specified* (Fithian, Kelly & Mow, 1990)	Human (ACL, PCL, MCL, LCL) 16–97 years*
		Bovine (distal femur and patella)			
		Some information untraceable (Lotz, Gerhart & Hayes, 1991; Reilly & Burstein, 1975; Mente & Lewis, 1994)	Some information untraceable (Repo & Finlay, 1977)		Some information untraceable (Trent, Walker & Wolf, 1976; Noyes & Grood, 1976; Woo et al., 1991)
Peña et al. (2005)	Compare stresses on menisci and cartilage healthy joints to meniscal tears and meniscectomies under compression up to 1,150 N	N/a	Information untraceable	Canine (menisci) (LeRoux & Setton, 2002)	Theoretical data (Weiss & Gardiner, 2001)
Peña et al. (2006)	Ligament and Menisci behaviour in healthy during compressive load transmission up to 1,150 N	N/a	Information untraceable	Canine (menisci) (LeRoux & Setton, 2002)	Human (ACL, PCL, MCL, LCL) 37–74 years* (Butler, Kay & Stouffer, 1986; Gardiner & Weiss, 2003; Blankevoort, Huiskes & De Lange, 1988***; Brantigan & Voshell, 1941***; Butler et al., 1990)
Donlagic et al. (2008)	Simulated knee joint kinematics during flexion	Human (proximal femur and mid femur) years*	Human (tibial plateau) age not specified*	Human (menisci) age not specified* (Fithian, Kelly & Mow, 1990)	Human (ACL, PCL, MCL, LCL) 16–97 years*
		Bovine (distal femur and patella)	Bovine (femoral condyle and tibial plateau)		Some information untraceable (Trent, Walker & Wolf, 1976; Noyes & Grood, 1976; Woo et al., 1991)
			Porcine (femoral condyle and tibial plateau)		
		Some information untraceable (Lotz, Gerhart & Hayes, 1991; Reilly & Burstein, 1975; Mente & Lewis, 1994)	Some information untraceable (Repo & Finlay, 1977; Laasanen, 2003)		
Shirazi, Shirazi-Adl & Hurtig (2008)	Role of collagen fibrils under compression up to 2,000 N	N/a	Human (tibial plateau) 48–70 years (Hayes & Mockros, 1971)	Human (menisci) 29–45 years	Human (ACL, PCL, LCL) 53–98 year* (Butler, Kay & Stouffer, 1986; Race & Amis, 1994)
				Some information untraceable (Tissakht & Ahmed, 1995)	
Guo, Zhang & Chen (2009)	Cartilage contact pressures during the gait cycle	Information untraceable	Information untraceable	Canine (menisci) (LeRoux & Setton, 2002)	Information untraceable
Yang et al. (2010)	Tibiofemoral angle effect on cartilage pressure during stance phase of gait	N/a	Information untraceable**	Information untraceable	Human (ACL, PCL, LCL) 43–74 years
					Some information untraceable (Butler, Kay & Stouffer, 1986; Blankevoort, Huiskes & De Lange, 1988***)
Kazemi et al. (2011)	Creep behaviour of cartilage and menisci under 300 N compression in healthy	N/a	Bovine (humeral head) (Langelier & Buschmann, 1999; Woo, Akeson & Jemmott, 1976)	Human (menisci) 29–45 years (Tissakht & Ahmed, 1995)	Human (patella tendon, Achilles tendon) 29–93 years; Rat (tail tendon) (Hansen et al., 2006; Johnson et al., 1994; Louis-Ugbo, Leeson & Hutton, 2004; Ault & Hoffman, 1992a)
Wang, Fan & Zhang (2014)	Cartilage stress during kneeling and standing with up to 1,000 N compression	Human (tibial plateau and femoral neck) 53–93 years* (Rho, Ashman & Turner, 1993; Zysset et al., 1999)	Human (femoral condyle and tibial plateau) 33–80 years (Shepherd & Seedhom, 1999a)	Human (menisci) 29–45 years*	Human (ACL, PCL, LCL, quadriceps tendon, patella ligament) 24–98 years*
				Bovine (menisci)	Some information untraceable (Butler, Kay & Stouffer, 1986; Race & Amis, 1994; Staubli et al., 1999; Blankevoort, Huiskes & De Lange, 1988***; Brantigan & Voshell, 1941***)
				Some information untraceable (Tissakht & Ahmed, 1995; Skaggs, Warden & Mow, 1994)	
Mootanah et al. (2014)	Joint forces/pressures due to malalignment with axial loads of 374 N	Human (femoral condyle and tibial plateau) 45–68 years (Hobatho et al., 1991)	Human (femoral condyle and tibial plateau) 33–80 years (Shepherd & Seedhom, 1997; Blankevoort, Huiskes & De Lange, 1988***)	Information untraceable	Human (ACL, PCL, MCL, LCL) 50 years primary data
Kazemi & Li (2014)	Viscoelastic poromechanical response of cartilage and menisci with compression up to 700 N	N/a	Human (tibial plateau) 48–70 years	Human (menisci) 29–45 years (Tissakht & Ahmed, 1995)	Human (ACL, PCL, LCL, patella tendon, Achilles tendon) 29–98 years*
			Bovine (humeral head) (Langelier & Buschmann, 1999; Woo, Akeson & Jemmott, 1976; Hayes & Mockros, 1971)		Rat (tail tendon) (Butler, Kay & Stouffer, 1986; Race & Amis, 1994; Blankevoort, Huiskes & De Lange, 1988***; Brantigan & Voshell, 1941***; Hansen et al., 2006; Johnson et al., 1994; Louis-Ugbo, Leeson & Hutton, 2004; Ault & Hoffman, 1992a)

**Notes:**

Summary of recent FE models of whole human knee joints and the type of sample each original primary data collection was based on including location of sample, and age if human samples were used.

ACL, anterior cruciate ligament; PCL, posterior cruciate ligament; MCL, medial collateral ligament; LCL, lateral collateral ligament.

* Age not specified in original research article.

** Multiple references are available in cited reference—unclear as to which study the FE model is using.

*** Material properties are not represented—papers are referenced with use of geometry and orientation of structure.

**Figure 5:**
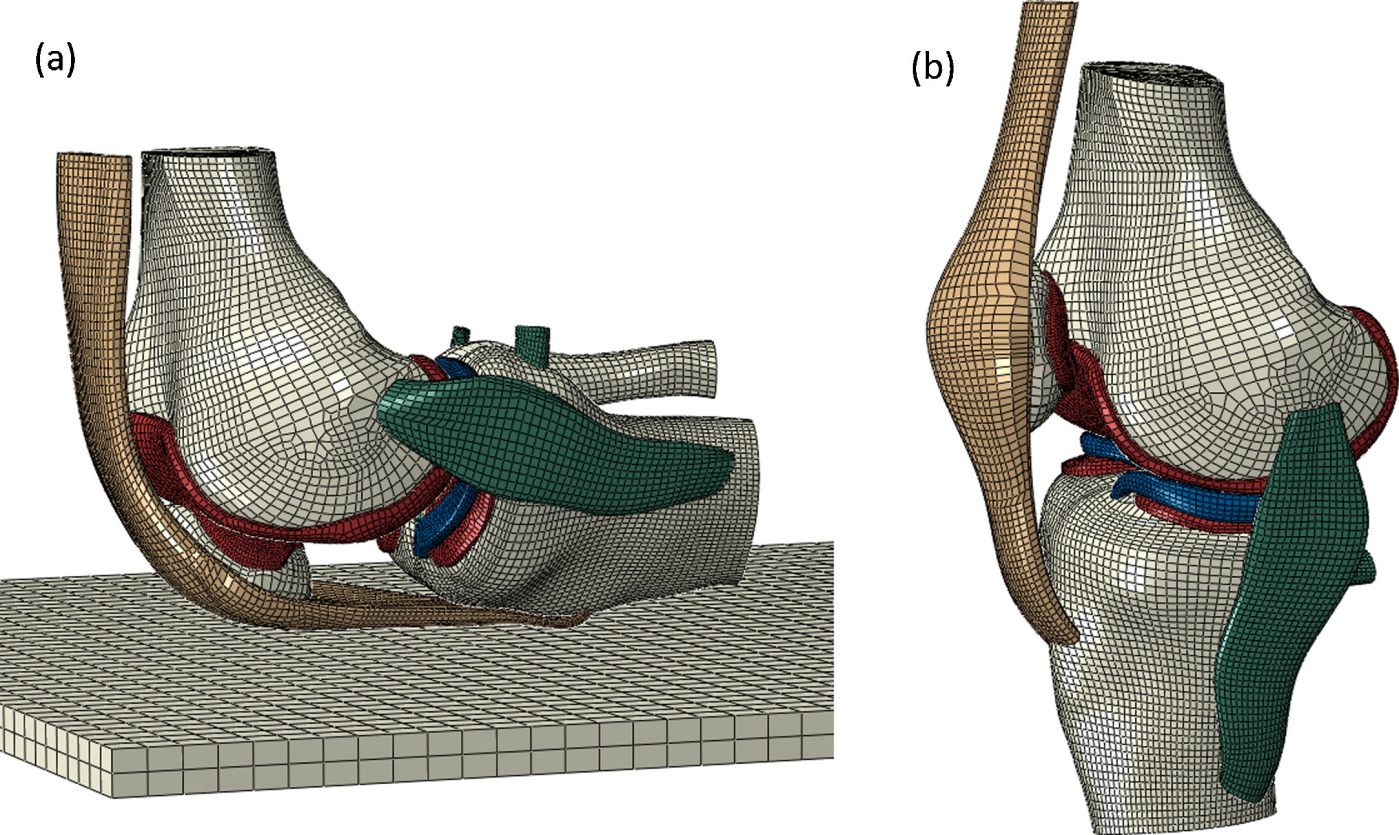
A finite element model of the knee joint. A FE model of the knee joint in (A) Kneeling position and (B) standing position. All structures are modelled in three dimension including the distal femur, proximal tibia and patella bones, femoral and tibial cartilage, medial and lateral menisci, ACL (anterior cruciate ligament), PCL (posterior cruciate ligament), MCL (medial collateral ligament), LCL (lateral collateral ligament) and patella tendon. (Reused from Wang, Fan & Zhang (2014): Elsevier License Permission: 4226550209690).

Wang, Fan & Zhang (2014) attempted to estimate cartilage stress under forces incurred during kneeling in a young healthy male (26-year-old), using primary MRI data to create their FE model, which it should be noted included the patella (Fig. 5). The referencing chain starting from Wang, Fan & Zhang (2014) follows up to five secondary references until the original research article is cited. Original demographics include human tibial plateau and femoral neck samples for bone (Rho, Ashman & Turner, 1993; Zysset et al., 1999), human femoral condyle and tibial plateau samples for cartilage (Shepherd & Seedhom, 1999a), human (Tissakht & Ahmed, 1995) and bovine menisci (Skaggs, Warden & Mow, 1994) and human ACL, PCL, LCL, quadriceps tendon and patella ligament samples for ligament material properties (Race & Amis, 1994; Woo et al., 1991; Staubli et al., 1999; Blankevoort, Huiskes & De Lange, 1988; Brantigan & Voshell, 1941). Where human samples were used for bone material properties the original research articles either do not state donor age (Rho, Ashman & Turner, 1993) or donor age was 53–93 years old (Zysset et al., 1999). Human cartilage ranged from 33 to 80 years old (Shepherd & Seedhom, 1999a) whilst menisci was either 29–45 years old (Skaggs, Warden & Mow, 1994) or information was not available. Human ligament samples had an average age of 24.9 years old (Staubli et al., 1999), an age range of 53–98 years old (Race & Amis, 1994), 43–74 years old (Blankevoort, Huiskes & De Lange, 1988), or it stated that donors were ‘young’ (Butler, Kay & Stouffer, 1986) or it was unspecified (Brantigan & Voshell, 1941) (Table 4). The specific material properties used within Wang, Fan & Zhang (2014), can be found in the Table 5 alongside the material properties from other FE modelling studies reviewed.

**Table 5:** Summary of material properties included in finite element models.

	Bone		Cartilage		Menisci		ACL		PCL		MCL		LCL	
	*E* (MPa)	*v*	*E* (MPa)	*v*	*E* (MPa)	*v*	*E* (MPa) or stiffness (N)	Initial strain (%/mm)	*E* (MPa) or stiffness (N)	Initial strain (%/mm)	*E* (MPa) or stiffness (N)	Initial strain (%/mm)	*E* (MPa) or stiffness (N)	Initial strain (%/mm)
Wang, Fan & Zhang (2014)	20,000	0.3	10	0.05–0.45	20–140	0.2	NS	0.0–0.1%	NS	0.0–0.1%	NS	0.0–0.1%	NS	0.0–0.1%
Peña et al. (2006)	Rigid	Rigid	5	0.46	59	0.49	1.95 MPa	0.0–0.1%	3.25 MPa	0.0–0.1%	1.44 MPa	0.0–0.1%	1.44 MPa	0.0–0.1%
Peña et al. (2005)	Rigid	Rigid	5	0.46	59	0.49	5.83 MPa	NS	6.06 MPa	NS	6.43 MPa	NS	6.06 MPa	NS
Guo, Zhang & Chen (2009)	11,000	0.3	5	0.45	59	0.46	NS	NS	NS	NS	NS	NS	NS	NS
Mootanah et al. (2014)	1,000	0.3	25	0.45	20–120	0.2–0.3	154 MPa	NS	40 MPa	NS	43 MPa	NS	56 MPa	NS
Kazemi et al. (2011)	Rigid	Rigid	0.26–1,600	0.36	0.5–28	0.36	10–14,000 MPa	NS	10–14,000 MPa	NS	10–14,000 MPa	NS	10–14,000 MPa	NS
Kazemi & Li (2014)	Rigid	Rigid	0.41–367.14	NS	0.0–12.84	NS	46.47–1,118.6 MPa	2.5%	46.47–1,118.6 MPa	0%	46.47–1,118.6 MPa	2%	46.5–1,118.6 MPa	2%
Donlagic et al. (2008)	1,000	0.3	67.6	0.3	130	0.3	200–260 MPa	NS	200–260 MPa	NS	114–134 MPa	NS	114–134 MPa	NS
Li, Lopez & Rubash (2001)	Rigid	Rigid	3.5–10	0.45	NM	NM	5,000 N	0.3–0.8 mm	9,000 N	2.3–3 mm	2,750 N	0.2–0.4 mm	2,000 N	−0.4 mm
Li et al. (1999)	Rigid	Rigid	5	0.45	NM	NM	5,000 N	0.3–0.8 mm	9,000 N	2.3–3 mm	2,750 N	0.2–0.4 mm	2,000 N	−0.4 mm
Blankevoort et al. (1991)	Rigid	Rigid	5	0.45	NM	NM	5,000 N	0.06–0.1%	9,000 N	−0.03 to −0.24%	2,750 N	0.03–0.04%	2,000 N	−0.05 to −0.25%
Blankevoort & Huiskes (1991)	Rigid	Rigid	5	0.45	NM	NM	5,000 N	0.06–0.1%	9,000 N	−0.03 to −0.24%	2,750 N	0.03–0.04%	2,000 N	−0.05 to −0.25%
Bendjaballah, Shirazi-Adl & Zukor (1995)	Rigid	Rigid	12	0.45	8–15	0.45	NS	1.2–4%	NS	−1 to −16.9%	NS	1.8–3.4%	NS	2.6–5%
Bendjaballah, Shirazi-Adl & Zukor (1997)	Rigid	Rigid	12	0.45	8–15	0.45	NS	1.2–4%	NS	−1 to −16.9%	NS	1.8–3.4%	NS	2.6–5%
Bendjaballah, Shirazi-Adl & Zukor (1998)	Rigid	Rigid	12	0.45	8–15	0.45	NS	1.2–4%	NS	−1 to −16.9%	NS	1.8–3.4%	NS	2.6–5%
Jilani, Shirazi-Adl & Bendjaballah (1997)	Rigid	Rigid	12	0.45	8–15	0.45	NS	1.2–4%	NS	−1 to −16.9%	NS	1.8–3.4%	NS	2.6–5%
Moglo & Shirazi-Adl (2003)	Rigid	Rigid	12	0.45	8–15	0.45	NS	1.2–4%	NS	−1 to −16.9%	NS	1.8–3.4%	NS	2.6–5%
Shirazi, Shirazi-Adl & Hurtig (2008)	Rigid	Rigid	12	0.45	8–15	0.45	NS	1.2–4%	NS	−1 to −16.9%	NS	1.8–3.4%	NS	2.6–5%
Yang et al. (2010)	Rigid	Rigid	15	0.45	20–140	0.2–0.3	5,000 N	0.06–0.1%	9,000 N	−0.03 to −0.24%	2,750 N	0.03–0.04%	2,000 N	−0.05 to −0.25%
Beillas et al. (2004)	75–17,500	0.3	20	0.45	250	0.45	150 MPa	NS	150 MPa	NS	60 MPa	NS	60 MPa	NS

**Notes:**

Material property values included in each of the finite element modelling studies.

*E,* elastic modulus; *v,* Poisson’s ratio; NM, not modelled; NS, not specified; ACL, anterior cruciate ligament; PCL, posterior cruciate ligament; MCL, medial collateral ligament; LCL, lateral collateral ligament.

Consecutive studies by Peña et al. (2005, 2006) carried out FE modelling of a healthy knee joint using CT and MRI data of a healthy male volunteer (age not specified) to generate a model that included bone, ligaments, tendons and articular and meniscal cartilages using previously published material property data. The aims of these studies were to compare stress and strain in a healthy human knee to those experienced after meniscal tears and meniscectomies (Peña et al., 2005) and to analyse the non-uniform stress–strain fields that the menisci and ligaments encounter during the loading of the human knee joint (Peña et al., 2006). The referencing chain starting from Peña et al. (2006) also follows up to four secondary references until the original research article is cited. As bones were modelled as rigid this requires no material property input; cartilage material properties could not be traced; menisci material properties were based on canine meniscal material properties (LeRoux & Setton, 2002) and ligaments on human ACL, PCL, MCL and LCL material properties with ages specified as 38 years old (Butler et al., 1990), 37–61 years old (91), 43–74 years old (Blankevoort, Huiskes & De Lange, 1988) or simply denoted as ‘young’ (Butler, Kay & Stouffer, 1986) or unspecified (Brantigan & Voshell, 1941). Peña et al. (2005) used the same original sources for cartilage and menisci material properties and adopted ligament material property data from a review article (Weiss & Gardiner, 2001) for the representation of a healthy knee joint, summarised in Table 4.

Guo, Zhang & Chen (2009) created a 3D human knee joint model from a CT scan on a 45-year-old healthy female to understand the contact pressures on the femoral and tibial cartilages during different phases of the gait cycle. Material properties were referenced from previous FE modelling papers; however, the referencing chain provides information that menisci data was originally presented by LeRoux & Setton (2002) based on canine meniscal properties. Unfortunately, bone, cartilage and ligament material property sources cannot be traced back to a primary data collection reference (Table 4).

A recent FE study explored misalignment differentiation of the knee joint to understand how this influences contact pressure (Mootanah et al., 2014). An MRI of a 50-year-old cadaveric male was used for geometry and validation of the model through mounting the knee joint and matching loading and boundary conditions. Mootanah et al. (2014) obtained material properties from the literature with a referencing chain going back through three other research papers to the original primary research article. Bone material properties were based on human femoral condyle and tibial plateau samples aged 45–68 years old (Hobatho et al., 1991) whilst cartilage was based on ages stated as 33–80 years old (Shepherd & Seedhom, 1997, 1999b). It is unclear how the meniscal material properties were obtained. Ligament material property data was obtained through primary data collection of the ACL, PCL, MCL and LCL giving validated values for the geometry of the FE model (Table 4).

Kazemi et al. (2011) used a MRI scan of a healthy 26-year-old male to construct an FE model to understand the differences in creep behaviour of intact knee joints that have undergone meniscectomies. Subsequent research by Kazemi & Li (2014) similarly used an MRI of a healthy 27-year-old male, and modelled structures with the same modelling theories as Kazemi et al. (2011), although marginally adapted these material property inputs in order to understand the poroelastic response of soft tissues in the knee joint under large compression forces. Original data collection for material properties used within both studies was derived from bovine humeral head cartilage (Langelier & Buschmann, 1999; Woo, Akeson & Jemmott, 1976) and human tibial plateau (29–45 years old) along with human menisci (Tissakht & Ahmed, 1995). However ligament material properties, specifically toe region fibril data, were based on previous studies of the human patella tendon aged 29–93 years old (Hansen et al., 2006; Johnson et al., 1994) and human calcaneal (Achilles) tendon aged 57–93 years old (Louis-Ugbo, Leeson & Hutton, 2004). The non-fibril ligament material properties can be traced back to a theoretical modelling paper (Ault & Hoffman, 1992a), whose results are represented in a companion paper with experimental work carried out on a rat tail tendon (Ault & Hoffman, 1992b). Ligament initial strains used within Kazemi & Li (2014) can be traced back to Peña et al. (2006) which as discussed previously are originally sourced from human specimens aged 43–74 years old (Blankevoort, Huiskes & De Lange, 1988), 53–98 years old (Race & Amis, 1994), or ages are described as ‘young’ (Butler, Kay & Stouffer, 1986) or unspecified (Brantigan & Voshell, 1941) (Table 4).

### Simplified FE models of the healthy human knee joint

For computational simplicity FE models of a human knee joint often make adjustments to their model including representing ligaments as non-linear one dimensional springs (Li, Lopez & Rubash, 2001; Blankevoort & Huiskes, 1991; Blankevoort et al., 1991; Li et al., 1999; Donlagic et al., 2008), bones as rigid bodies lacking material properties (Li, Lopez & Rubash, 2001; Li et al., 1999; Bendjaballah, Shirazi-Adl & Zukor, 1995; Jilani, Shirazi-Adl & Bendjaballah, 1997; Shirazi, Shirazi-Adl & Hurtig, 2008) or exclusion of particular structures such as the menisci (Blankevoort & Huiskes, 1991; Blankevoort et al., 1991) or ligaments (Guess et al., 2010; Donahue et al., 2002, 2003).

Models that have been highly simplified but still integrate all the main structures of the knee joint include studies by Blankevoort et al. (1991) and Blankevoort & Huiskes (1991) who created mathematical models of the knee joint, developed originally by Wismans et al. (1980), specifically focusing on the articular contact and interaction between ligaments and bones. Utilising the previously developed modelling theories (Blankevoort & Huiskes, 1991; Blankevoort et al., 1991). Li et al. (1999) and Li, Lopez & Rubash (2001) used a MRI of a 65-year-old male cadaver to create a 3D model of the knee joint and conducted a sensitivity analysis varying input parameters to assess the effect on joint contact stresses. In continuation, Yang et al. (2010) also utilised the work proposed by Blankevoort et al. (1991) and Blankevoort & Huiskes (1991) to define MRI scans from three young volunteers (21–23 years old) to determine cartilage contact stress during gait; however, noticeable differences between studies include the representation of the menisci within Yang et al. (2010).

Within these corresponding studies ligaments were modelled as ‘bars,’ which are one-dimension (1D) non-linear tension-only elements with just two nodes, although material properties are still assigned. It should also be noted that Li, Lopez & Rubash (2001) stated that ligament stiffness was optimised for the model to ensure numerical stability and model convergence rather than utilising a value measured experimentally. Blankevoort et al. (1991), Blankevoort & Huiskes (1991), Yang et al. (2010), Li et al. (1999) and Li, Lopez & Rubash (2001) sourced ligament material properties from human ACL, PCL and LCL samples aged ‘young’ (Butler, Kay & Stouffer, 1986) or aged 43–74 years old (Blankevoort, Huiskes & De Lange, 1988). Unfortunately, cartilage material properties were ambiguous due to multiple references available in the cited sources (Kempson, 1980; Mow, Lai & Holmes, 1982) making the origin of the input data unclear. Additionally, the menisci were modelled within Yang et al. (2010); however, the original data collection reference could not be traced. Referencing information from these FE studies are summarised in Table 4.

**Figure 6:**
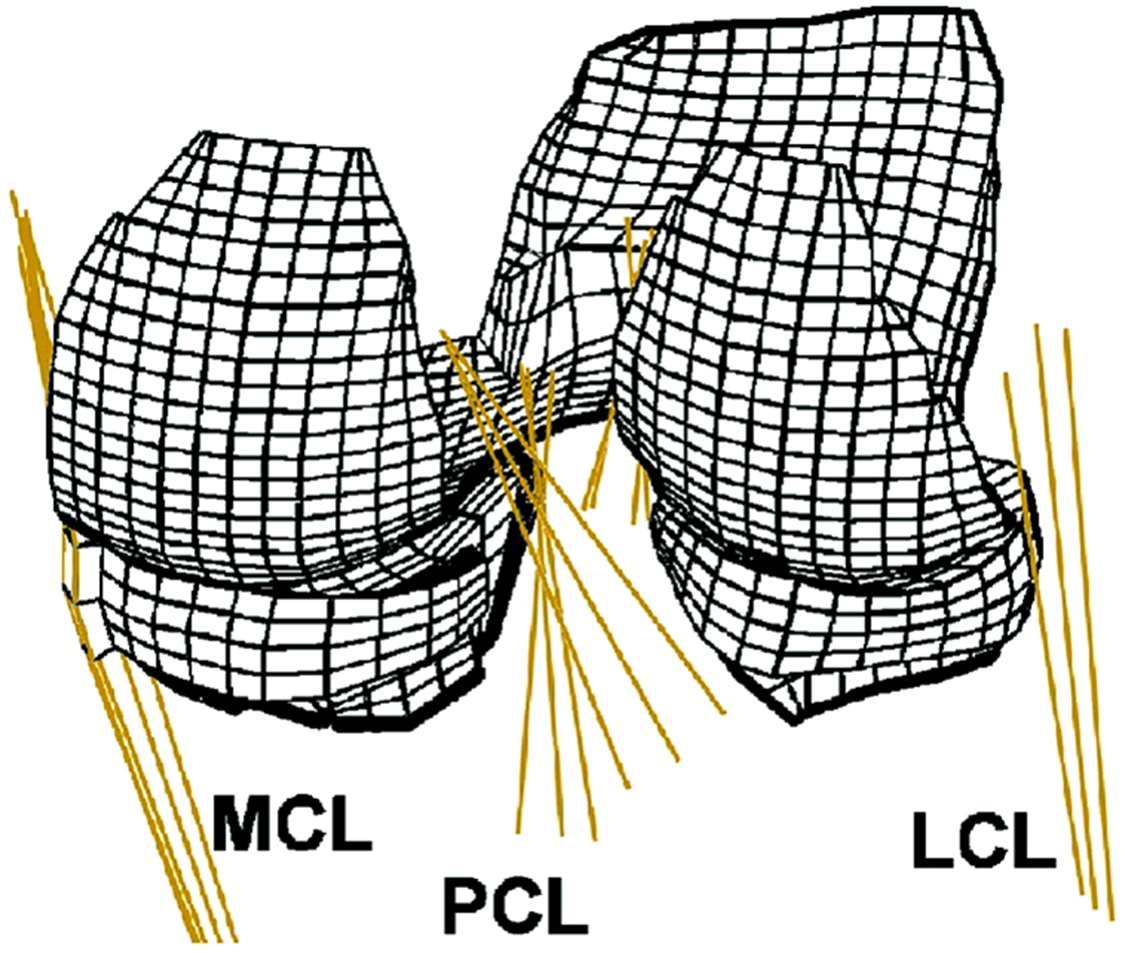
Human knee finite element mesh. Posterior view of a finite element mesh showing soft tissues (menisci and articular cartilage layers). Ligaments are modelled as one dimensional line elements. Rigid bodies representing the femur and the tibia are not shown. (Adapted from Shirazi, Shirazi-Adl & Hurtig (2008): Elsevier License Number: 4226550481987).

In addition to simplifying anatomical geometry it is also common for investigators to reuse medical image data sets to create different models. In sequential studies CT data of a 27-year-old female was used to construct a FE model of the human knee joint to explore contact pressures (Bendjaballah, Shirazi-Adl & Zukor, 1995), varus and valgus alignment (Bendjaballah, Shirazi-Adl & Zukor, 1997), axial rotation (Jilani, Shirazi-Adl & Bendjaballah, 1997), anterior–posterior forces (Bendjaballah, Shirazi-Adl & Zukor, 1998), ACL and PCL coupling (Moglo & Shirazi-Adl, 2003) and cartilage collagen fibril response to compression (Shirazi, Shirazi-Adl & Hurtig, 2008). Figure 6 illustrates the model created within these studies and highlights the differences in comparison to Fig. 5 in mesh generation and inclusion of all structures in 3D form. When tracing the material properties assigned to structures within these corresponding FE models cartilage primary data was ascertained from human tibial plateau samples aged 48–70 years old (Hayes & Mockros, 1971), ligaments from human ACL, PCL and LCL samples, referenced with ages of 53–98 years old (Race & Amis, 1994), or from samples described as ‘young’ (Butler, Kay & Stouffer, 1986). Menisci material properties were based on human meniscal samples aged 29–45 years old (Tissakht & Ahmed, 1995) alongside additional data which could not be traced (Table 4).

Another simplified FE model was developed by Beillas et al. (2004) who modelled the whole lower limb of a 30-year-old male and coordinated this with in vivo kinematics of a one-leg hop. However, this model was simplified with a 1D representation of the ligaments. Bone material properties were originally obtained from proximal femur and mid femur human samples aged either 28–91 years old (Lotz, Gerhart & Hayes, 1991), or age was unspecified (Reilly & Burstein, 1975), or bovine samples were used (Mente & Lewis, 1994). Cartilage material properties can be traced to human tibial plateau samples although age was not specified (Repo & Finlay, 1977) and some further cartilage information was untraceable. Menisci data also came from human samples although again age was not specified (Fithian, Kelly & Mow, 1990). Finally, ligament material properties were based on human ACL, PCL, MCL and LCL data obtained from donors aged 16–86 years old (Noyes & Grood, 1976), 29–55 years old (Trent, Walker & Wolf, 1976), and 22–97 years old (Woo et al., 1991) (Table 4).

Incorporating some of the material properties presented by Beillas et al. (2004), Donlagic et al. (2008) utilised a patient specific approach to derive geometry and loads for their FE model using an MRI of a 22- and 52-year-old male alongside primary kinematic data of flexion and extension locomotion. However, additional material property sources were also used for the representation of the cartilage including bovine and porcine femoral condyle and tibial plateau samples (Laasanen, 2003) (Table 4).

### FE models of OA human knee joints

It was discussed previously (Section A—Material Properties, above) that changes in tissues structure during OA progression can result in changes in material properties. This in turn would correlate with a change in the response to loads and biomechanics of the whole knee joint. With this in mind, FE modelling has the potential to analyse such alterations in the presence of OA, assuming that tissue material properties representative of diseased tissues are incorporated into models. Although some FE studies have attempted to investigate contact stresses to understand how OA can initiate and progress (Peña et al., 2007b; Dong et al., 2011; Mononen et al., 2011, 2012, 2016; Venäläinen et al., 2016) or how arthroplasty procedures can affect the knee joint (Baldwin et al., 2012; Tuncer et al., 2013) there is only a handful of research papers that utilise a whole knee joint FE model based specifically on healthy versus OA material properties.

One of the first studies to attempt this examined how osteochondral defects influence the ongoing degeneration and stress concentrations of cartilage in the knee joint during compression based on the geometry and anatomical location of the defect (Peña et al., 2007b). Healthy material properties were identical to Peña et al. (2006) described in detail above and therefore included human and canine tissue. However, when modelling cartilage with defects the elastic modulus of the cartilage was adjusted to 1.5 MPa with data originally sourced from Athanasiou et al. (1995) who explored the elastic modulus of rabbit cartilage with artificially induced OA. A similar study by Dong et al. (2011) also explored the cartilage defects but kept the elastic modulus consistent for both healthy and OA simulations.

Although not modelling a whole knee, consecutive studies by Mononen et al. (2011, 2012) segmented the femoral and tibial cartilage from 29- and 61-year-old healthy males for FE analysis modelling the cartilage with fibril-reinforced poroviscoelastic properties. Mononen et al. (2011) compared normal, OA and repaired cartilage giving a strain dependent fibril network modulus of 673, 168 and 7–505 MPa respectively; an initial fibril network modulus of 0.47, 0.47 and 0.005–0.35 MPa respectively; an elastic modulus of 0.31, 0.08 and 0.31 MPa respectively; and finally a Poisson’s ratio of 0.42 for all samples. Mononen et al. (2012) compared only normal and OA samples with the same material properties. When following the referencing chain and tracing cartilage material properties back to their original research they used input data from bovine articular cartilage (DiSilvestro & Suh, 2001; Korhonen et al., 2003) where OA was artificially induced (Korhonen et al., 2003).

## Discussion

### Material properties

There is considerable variation in the elastic modulus of articular cartilage obtained from the human knee joint within the literature. This can be at least attributed to differences in testing parameters and structure and quality of the tissue sample, in addition to known and ambiguous variation in donor characteristics. To summarise, samples within the literature include hydrated (Wilusz, Zauscher & Guilak, 2013; Kleemann et al., 2005; Hori & Mockros, 1976; Franz et al., 2001; Wang et al., 2013; Shepherd & Seedhom, 1997, 1999a) and dehydrated (Wen et al., 2012) femoral and tibial localities and ages between 32 and 89 years old. Furthermore OA samples have been graded using the Collins (Collins, 1939, 1949 cited in Wilusz, Zauscher & Guilak (2013)), Bollet (Bollet, Handy & Sturgill, 1963 cited in Hori & Mockros (1976)) and Outerbridge (Outerbridge, 1961) scoring systems, creating inconsistencies in categorisation. Both confined and unconfined compression testing has been employed (Kleemann et al., 2005; Hori & Mockros, 1976; Thambyah, Nather & Goh, 2006) alongside indentation techniques (Franz et al., 2001; Shepherd & Seedhom, 1997, 1999a) and AFM (Wen et al., 2012; Wilusz, Zauscher & Guilak, 2013; Wang et al., 2013). Research also incorporates extensive ranges in testing specifications including indentation tip radius (10–30.4 mm) (Hori & Mockros, 1976; Wen et al., 2012; Franz et al., 2001; Shepherd & Seedhom, 1997, 1999a; Thambyah, Nather & Goh, 2006; Wilusz, Zauscher & Guilak, 2013; Wang et al., 2013), loading force (0.019–11.8 N) (Kleemann et al., 2005; Hori & Mockros, 1976) and recovery phases if included (5 min) (Thambyah, Nather & Goh, 2006).

As discussed in ‘Section A—Material Properties,’ length scale dependency can affect the values derived from testing. For example, heterogeneity can be more easily identified in cartilage using nanoindentation when compared to microindentation (Stolz et al., 2009, 2004), which is particularly important when changes due to OA can be subtle. When reviewing current efforts at measuring elastic modulus of human knee joint cartilage, variation will indeed exist due to differing length scales between 10 nm (Wen et al., 2012) and 30.4 mm (Hori & Mockros, 1976) which may have an effect on obtained modulus. Moreover, studies also present varying elastic modulus, namely instantaneous (Franz et al., 2001; Hori & Mockros, 1976; Shepherd & Seedhom, 1997; Shepherd & Seedhom, 1999a; Thambyah, Nather & Goh, 2006; Wilusz, Zauscher & Guilak, 2013) and equilibrium modulus with some citing a 30 s (Wen et al., 2012) to 10 min (Kleemann et al., 2005) hold period. The circumstances under which tissues are measured will influence the results, and therefore the ability to compare across studies and accurately apply such data in FE models. It has previously been shown that there are considerable differences in instantaneous and equilibrium modulus, where instantaneous produces a much higher value (Julkunen et al., 2009), highlighting the need for a more standardised method of testing to determine any subtle change in material properties during healthy ageing and OA that may not be comparable across multiple data sources.

With these variations in mind elastic modulus for hydrated healthy cartilage samples varies between 0.1and 18.6 MPa (Wilusz, Zauscher & Guilak, 2013; Thambyah, Nather & Goh, 2006; Brittberg & Peterson, 1998; Bae et al., 2003; Shepherd & Seedhom, 1997, 1999a), hydrated OA grade 1 samples range between 0.5 and 10.2 MPa (Kleemann et al., 2005; Hori & Mockros, 1976; Franz et al., 2001; Wang et al., 2013) and hydrated OA grade 2 and 3 between 0.1 and 0.5 MPa (Wilusz, Zauscher & Guilak, 2013; Kleemann et al., 2005; Wang et al., 2013), noting that different OA grading systems are used across these studies. Furthermore, age ranges stated within the literature have a wide variation, the broadest being 33–80 years old within one study (Shepherd & Seedhom, 1999a). Some values cannot be explicitly linked to age ranges. Future work is required to more definitely define changes in cartilage material properties associated to explicitly with age and therefore help understand how alterations through disease can be separated from alterations during healthy ageing.

In comparison to the available data on human knee joint cartilage, there is significantly less data for femoral or tibial bone samples. Indeed, this research found only one study that quantitatively measured material properties of cortical bone from the human knee joint (Rho, Tsui & Pharr, 1997). Data on trabecular properties is present but it is difficult compare data from different anatomical locations collected with different techniques, specifically traditional compression approaches (Lindahl, 1976; Goldstein et al., 1983; Burgers et al., 2008) and more recent nanoindentation methods (Rho, Tsui & Pharr, 1997), which is yet to be applied to the human femoral condyle. Similar ambiguity in the relationship between age and material properties also exists. Age ranges vary between 14 and 92 years old across studies with the smallest age cohort (with the exception of individual donors) spanning 20 years in one study (Goldstein et al., 1983). Some studies also used donors under the age of 30 where donors may not have reached skeletal maturity and material properties may not reflect peak bone mass (Matkovic et al., 1994). Overall, trabecular bone elastic modulus ranges from 1.9 MPa to 664.0 MPa across reviewed studies (Behrens, Walker & Shoji, 1974; Ducheyne et al., 1977; Carter & Hayes, 1977; Lindahl, 1976; Goldstein et al., 1983; Hvid & Hansen, 1985; Burgers et al., 2008; Zysset, Sonny & Hayes, 1994) and cortical bone from 22,500 MPa to 25,800 MPa (Rho, Tsui & Pharr, 1997).

Studies reviewed in ‘Section A—Material Properties’ mostly involve experimental work on trabecular bone which is less commonly used within FE models. Compression techniques utilised to obtain macroscale measurements of trabecular bone as a whole structure as opposed to measuring individual trabeculae, will inevitably produce lower elastic modulus values due to the nature of testing; however, more sophisticated techniques incorporating tissue level material properties can more accurately represent a structure such as trabecular bone at the level in which it is typically modelled in FE research (Nigg & Herzog, 2006). This variability in techniques inevitably makes a comparison between studies challenging as well as the lack of distinct age cohorts to ultimately define young and old parameters in order to definitively link this to a change in properties due to injury or disease, such as OA. Despite some research incorporating material properties of varying OA grades there are no healthy controls included to explicitly link significant findings to OA status (Zysset, Sonny & Hayes, 1994). Evidently there is also no material property data for human trabecular bone obtained from the distal femur or proximal tibia at the tissue level, comparing healthy and OA samples.

It should be noted that the studies cited herein utilised varying indenter sizes ranging from 20 nm (Rho, Tsui & Pharr, 1997) to 2.5 mm (Hvid & Hansen, 1985). A length scale under 200 nm is able to determine more heterogeneity in bone structure than those applied above 200 nm (Yao et al., 2011). When comparing studies discussed herein it should be considered that comparisons are challenging, and indeed reiterates the importance of site and subject-specific material properties, preferably obtained at the nanoscale to accurately present the human knee joint using FE modelling (Yao et al., 2011).

Likewise, there is also significant variation in ligament tensile properties reported in the literature and this could be attributed to a number of factors including the variation in cadaver cohorts, equipment and testing protocol and technique. Experimental procedures for ligament material properties vary between cross-sectional samples (Momersteeg et al., 1995) or bone–ligament–bone samples spanning a variety of age ranges with current data in the literature ranging from 16 to 97 years old (Harner et al., 1995; Quapp & Weiss, 1998; Butler et al., 1992; Robinson, Bull & Amis, 2005; Trent, Walker & Wolf, 1976; Noyes & Grood, 1976; Butler, Kay & Stouffer, 1986; Race & Amis, 1994; Woo et al., 1991; Chandrashekar et al., 2006). Preconditioning, which is often included as a ‘warm up’ for the ligament to achieve load-displacement parameters that are repeatable (Momersteeg et al., 1995) is absent from some research studies (Momersteeg et al., 1995; Noyes & Grood, 1976). Furthermore data varies across individual studies where elastic modulus of the knee ligaments ranges between 1.7 and 447.0 MPa (Quapp & Weiss, 1998; Butler et al., 1992; Noyes & Grood, 1976; Butler, Kay & Stouffer, 1986; Race & Amis, 1994; Chandrashekar et al., 2006) and failure load between 194.0 and 2160.0 N (Harner et al., 1995; Robinson, Bull & Amis, 2005; Trent, Walker & Wolf, 1976; Noyes & Grood, 1976; Race & Amis, 1994; Woo et al., 1991; Chandrashekar et al., 2006). Comparisons between young and old have been correlated for the ACL in two studies (Noyes & Grood, 1976; Woo et al., 1991) both concluding that young donors have a higher stiffness and failure load. However, this is yet to be explored in the PCL, MCL and LCL along with research into how ligament tensile properties are correlated to pathological existence in the form of OA.

### FE modelling

Finite elements models have been used for various applications involving the whole knee joint including healthy representation (Peña et al., 2006; Wang, Fan & Zhang, 2014), joint replacement mechanics (Baldwin et al., 2012; Tuncer et al., 2013), meniscectomy research (Tanska, Mononen & Korhonen, 2015), cartilage contact stresses (Li, Lopez & Rubash, 2001; Guo, Zhang & Chen, 2009) and ligament–bone interaction (Blankevoort et al., 1991) to name a few. Material properties used within the reviewed FE models are often sourced from the literature including previous modelling studies or primary experimental research. This typically results in highly variable data sets based on multiple structures and species. The material properties of human tissue vary according to its mineral and protein composition and the orientation of its micro-architecture (Wilusz, Zauscher & Guilak, 2013; Marticke et al., 2010; Temple-Wong et al., 2009). These factors in turn vary with anatomical location (e.g. femur vs humerus; knee vs ankle), age and health of the tissue. Therefore, donor characteristics will significantly impact results. It is clear that current whole joint FE models use material properties with highly variable, or non-specific material properties, with variation in the age, species, location and disease state of the tissue from which material properties were obtained.

When the values used for material properties within published FE models are traced to their original research citation it becomes clear that there is considerable variation in terms of age range. FE models produced by Beillas et al. (2004) and Donlagic et al. (2008) have a total age range across all structures of 16–97 years old. The smallest age range used for material properties within a single study is 43–74 years old (Li, Lopez & Rubash, 2001; Blankevoort & Huiskes, 1991; Blankevoort et al., 1991; Li et al., 1999; Yang et al., 2010), with other ages ranging between 37 and 74 years old (Peña et al., 2005), 33–80 years old (Mootanah et al., 2014), 29–93 years old (Kazemi & Li, 2014), 29–98 years old (Kazemi & Li, 2014; Bendjaballah, Shirazi-Adl & Zukor, 1995; Jilani, Shirazi-Adl & Bendjaballah, 1997; Shirazi, Shirazi-Adl & Hurtig, 2008; Bendjaballah, Shirazi-Adl & Zukor, 1997, 1998; Moglo & Shirazi-Adl, 2003) and 25–98 years old (Wang, Fan & Zhang, 2014). In many FE modelling studies, some information including age of donors from the original sources of material properties could not be traced (Peña et al., 2005, 2006; Wang, Fan & Zhang, 2014; Li, Lopez & Rubash, 2001; Guo, Zhang & Chen, 2009; Mootanah et al., 2014; Kazemi & Li, 2014; Blankevoort & Huiskes, 1991; Blankevoort et al., 1991; Li et al., 1999; Donlagic et al., 2008; Bendjaballah, Shirazi-Adl & Zukor, 1995, 1997, 1998; Jilani, Shirazi-Adl & Bendjaballah, 1997; Shirazi, Shirazi-Adl & Hurtig, 2008; Yang et al., 2010; Moglo & Shirazi-Adl, 2003; Beillas et al., 2004). Where material properties are categorised by age there are considerable differences between cohorts, most noticeably in ligament data (Noyes & Grood, 1976; Woo et al., 1991). In particular Woo et al. (1991) recorded the site of failure in ligaments when loaded in the anatomical location and concluded that in younger donors the ACL will predominantly fail by avulsion and in older donors the ACL will predominantly fail at the mid-substance, due to a change in material properties. This is especially important to factor into FE models if safety factors in the joint are being researched. The effect of using material properties from broad, and in some cases unknown age ranges, impacts on the conclusions of FE modelling is currently unclear because at present no study has compared these models to one constructed using anatomical geometry and material properties for all tissues from the same individual, or a homogeneous age and gender cohort of individuals. Such a model would clearly represent the ‘gold-standard’ with respect to geometry and material property definition in a FE knee model.

As well as wide variation in age, some FE models use material property data based just on tibial plateau cartilage (Kazemi & Li, 2014; Donlagic et al., 2008; Bendjaballah, Shirazi-Adl & Zukor, 1995, 1997, 1998; Jilani, Shirazi-Adl & Bendjaballah, 1997; Shirazi, Shirazi-Adl & Hurtig, 2008; Moglo & Shirazi-Adl, 2003; Beillas et al., 2004) or bone samples lacking any femoral condyle measurements (Wang, Fan & Zhang, 2014). Furthermore, they may be based on non-knee joint anatomical locations including femoral neck and mid femur bone material properties (Donlagic et al., 2008; Beillas et al., 2004) and humeral head for cartilage material properties (Kazemi et al., 2011; Kazemi & Li, 2014). As an example of the magnitude of disparity in material properties between different anatomical locations, Shepherd & Seedhom (1999a) tested the elastic modulus of ankle, knee and hip joint cartilage finding differences of up to 6.8 MPa (36.6%) between ankle and knee cartilage samples from the same donor and 3.6 MPa (30.54%) between knee and hip cartilage samples from the same donor. Indeed, it has been shown that variations in material properties from the same tissue exists within and across the knee joint suggesting that a location dependent modulus for various tissues would be most appropriate for FE models (Behrens, Walker & Shoji, 1974; Deneweth, Arruda & McLean, 2015; Akizuki et al., 1986). Thus, while better than using values from outside the knee joint itself, representing structures with homogeneous (i.e. only one value) properties, or for example, assuming tibial and femoral material properties are identical, may be sub-optimal and functionally important. Ligament material properties are also often replicated where original data is only based on selective ligaments of the knee joint (Wang, Fan & Zhang, 2014; Li, Lopez & Rubash, 2001; Kazemi & Li, 2014; Blankevoort & Huiskes, 1991; Blankevoort et al., 1991; Li et al., 1999; Bendjaballah, Shirazi-Adl & Zukor, 1995, 1997, 1998; Jilani, Shirazi-Adl & Bendjaballah, 1997; Shirazi, Shirazi-Adl & Hurtig, 2008; Yang et al., 2010; Moglo & Shirazi-Adl, 2003). In some instances tendon data is used for the representation of ligament material properties including the quadriceps tendon (Wang, Fan & Zhang, 2014), patella tendon (Wang, Fan & Zhang, 2014; Kazemi et al., 2011; Kazemi & Li, 2014), Achilles tendon (Kazemi et al., 2011; Kazemi & Li, 2014) and rodent tail tendon (Kazemi et al., 2011; Kazemi & Li, 2014).

Animal material property data is also commonly used in the representation of human knee FE models including bovine (Wang, Fan & Zhang, 2014; Mootanah et al., 2014; Shepherd & Seedhom, 1999b; Kazemi & Li, 2014; Donlagic et al., 2008; Beillas et al., 2004; Mononen et al., 2011, 2012), canine (Peña et al., 2005, 2006; Guo, Zhang & Chen, 2009), porcine (Donlagic et al., 2008), rat (Kazemi et al., 2011; Kazemi & Li, 2014) and rabbit (Peña et al., 2007b) data. A number of recent studies have highlighted the structural, mechanical and physiological differences between bovine and human soft tissue and questioned the suitability of bovine material property data for functional studies of humans (Demarteau et al., 2006; Jeffrey & Aspden, 2006; Nissi et al., 2007; Pedersen et al., 2013; Plumb & Aspden, 2005). Athanasiou et al. (1991) explored the differences between material properties of cartilage from the femoral condyle of different species and found variation between the Poisson’s ratio of human (0.074–0.098), canine (0.3–0.372), bovine (0.383–0.396) and rabbit (0.197–0.337) along with aggregate modulus of human (0.588–0.701 MPa), canine (0.603–0.904 MPa), bovine (0.894–0.899 MPa) and rabbit (0.537–0.741 MPa). Although differences were not statistically significant, potentially due to low samples numbers (*n* = 4–10) there was evidently a difference between species all of which have been used in some of the reviewed FE models. Further, it has also been shown that not only do material properties vary by species but they vary spatially within the same joint. For example, Peters et al. (2017) found differences of up to 10.5 MPa in elastic modulus of cartilage samples taken from different locations within a single canine knee joint. This can indeed have an effect on subsequent FE model behaviour predictions and should be taken into consideration where possible in future studies.

As discussed earlier, it is very common for FE modelling studies to source and reference their material property data from previous modelling studies rather than the original experimental studies in which practical measurements were obtained. However, when the referencing chain is followed through sequentially cited modelling papers it is often the case that the primary experimental source of material property data is untraceable (Yang et al., 2010; Peña et al., 2006). In other instances it eventually becomes clear that material property values are not source for direct experimental measures, but have been derived directly or indirectly from theoretical research in which mathematical solutions for modelling a specific structure have been derived (Mak, Lai & Mow, 1987 cited in Peña et al. (2005, 2006), Li, Lopez & Rubash (2001), Guo, Zhang & Chen (2009)).

Use of varying ages, species and anatomical locations for material property information undoubtedly represent important limitations in current FE models, but the magnitude of error is presently difficult to quantify and probably varies widely across studies due to the highly ‘mixed’ nature of input data used. At present, the best indication of error comes from studies that have conducted sensitivity analyses on material properties. Li, Lopez & Rubash (2001) conducted a sensitivity analysis varying cartilage elastic modulus from 3.5 MPa to 10 MPa and showed that peak contact stresses linearly increased by up to 10%, whilst an increase in Poisson’s ratio significantly varied peak von Mises stress by 100% in the knee joint cartilage. Additionally, a more sophisticated sensitivity analysis was carried out by Dhaher, Kwon & Barry (2010) who adjusted the intrinsic material properties of knee joint ligaments to aid understanding of the functional consequences of different activity levels, age, gender and even species. The research measured simulation outcomes by incorporating a multi-factorial global assessment, which indicated a change in tibial–femoral internal and external rotation, patella tilt and patella peak contact stresses, associated with modified ligament material properties (Dhaher, Kwon & Barry, 2010).

This review of published material property (Section A—Material Properties) and FE modelling (Section B: FE Modelling, above) studies of the human knee raises the question of how well specific cohorts or even human demographics can currently be accurately represented in a FE model. For example, does sufficient material property data exist to construct a whole-knee joint FE model representative of a young, healthy human or to represent a knee of any age with a specific category of OA? Attempting to build an FE model of a healthy knee joint from the literature data tabulated in ‘Section A—Material Properties’ (Tables 1–3) yields data for healthy femoral and tibial cartilage, although without the breakdown of age specific material properties; healthy tibial cortical bone from older donors; healthy ACL, PCL, MCL and LCL from young donors, and ACL, PCL and MCL from healthy older donors. Thus, ‘healthy’ material properties can be pieced together from different studies for most tissues but mixing gender and a considerable age range (16–97 years old) is necessary. In terms of a model for studying OA, data exists for cartilage material properties based on OA grades 1–3 although this is not broken down into age categories, whilst trabecular bone material properties do exist for OA grades 1–3 for older donors although challenges occur as no healthy control was used within this particular study as a baseline measurement. Further no study has yet explored the effect of OA on cortical bone material properties in the human knee. There is currently no data incorporating the effect of OA on ligament material properties despite it being well known that there is a relationship between OA and ligament injury (Mullaji et al., 2008; Cushner et al., 2003). However, there are currently no research papers to the authors’ knowledge that have collected primary data on bone and cartilage material properties and used these measurements to build a subject specific FE model. Hence, material properties are still collated from various sources within the literature. A key goal for future research should be adoption of a more subject specific approach in which material properties from all tissues are derived from homogenous donor cohorts to improve accuracy and precision of knee FE models.

## Conclusions and Future Directions

Integrating tissues-specific material property data into FE models has the potential to provide considerable insight into both healthy and diseased knee joint mechanics, circumventing the difficulty of direct invasive measures of human functionality. Herein, we have provided a comprehensive summation and evaluation of existing material property data for human knee joint tissues with all numerical values tabulated as a reference resource for future studies. A renaissance in material testing and engineering approaches in the last decade has yielded an abundance of data on the mechanical properties of both hard and soft tissues from the human knee joint. However, comparison of material properties between studies can be challenging due to the differences in cadaver age, data collection techniques, including orientation of the tissue and loading specifics (Chandrashekar et al., 2006). It is well documented that material properties alter during ageing (Hansen, Masouros & Amis, 2006), therefore the demographics of cadavers will highly influence material property data. Our review highlights that material properties from multiple (>1) tissue types have rarely been collected from cadavers with homogeneous age, gender and health status characteristics. More consistent data collection with particular emphasis on extracting data on multiple tissues from the same donors will enable a much more robust examination of the structural and mechanical changes occurring during ageing, injury and disease, notably during OA progression which currently represents a significant socio-economic burden that is likely to increase further within ageing populations.

The benefits of a more exhaustive subject- or cohort-specific approach to materials testing will inherently feed directly into improved FE models of whole-knee function. Efforts have been made to produce an openly available FE model for clinical and basic science research (Erdemir, 2016). With more accurate material property data from cohort specific sources data could be applied into this freely available model without the need to obtain medical imagery to create a new FE model which is costly in time and resources. More demographically homogenous material property data sets will eliminate the current widespread use of material properties sourced from distinctively diverse human cadavers and/or animal specimens. Embracing this more systematic subject- or cohort-specific approach to FE modelling can only improve comparisons between injured and diseased tissue within the knee joint, and enhance understanding of behavioural response to mechanical loads observed during ageing or disease progression. It is notable at present that no FE modelling study has compared healthy and OA whole-knee joints. Increasing ageing populations within western societies provide particular incentive for this research with a clear need to direct research efforts into better integration of mechanical engineering approaches and biomechanical simulation, particularly in the presence of disease status.

## Additional Information and Declarations

### Data Availability

The following information was supplied regarding data availability: The research in this article did not generate data or code as this is a literature review.
